# Clonal lineage tracing of innate immune cells in human cancer

**DOI:** 10.1016/j.ccell.2026.05.006

**Published:** 2026-06-04

**Authors:** Vincent Liu, Katalin Sandor, Patrick K. Yan, Zhuang Miao, Yajie Yin, Robert R. Stickels, Andy Y. Chen, Kamir Hiam-Galvez, Jacob Gutierrez, Wenxi Zhang, Sairaj M. Sajjath, Raeline Valbuena, Steven Wang, Bence Daniel, Leif S. Ludwig, Brooke E. Howitt, Caleb A. Lareau, Ansuman T. Satpathy

**Affiliations:** 1 Department of Genetics, Stanford University, Stanford, CA 94305, USA; 2 Department of Pathology, Stanford University, Stanford, CA 94305, USA; 3 Center for Immunotherapy Design, Stanford University, Stanford, CA 94305, USA; 4 Department of Bioengineering, Stanford University, Stanford, CA 94305, USA; 5 Computational and Systems Biology Program, Memorial Sloan Kettering Cancer Center, New York, NY 10065, USA; 6 Robin Chemers Neustein Laboratory of Mammalian Cell Biology and Development, Howard Hughes Medical Institute, The Rockefeller University, New York, NY 10065, USA; 7 Berlin Institute of Health at Charité – Universitätsmedizin Berlin, 10178 Berlin, Germany; 8 Max-Delbrück-Center for Molecular Medicine in the Helmholtz Association (MDC) Berlin Institute for Medical Systems Biology (BIMSB), 10115 Berlin, Germany; 9 Parker Institute for Cancer Immunotherapy, San Francisco, CA 94129, USA; 10 Lead contact

## Abstract

Innate immune cells constitute the majority of the tumor microenvironment (TME) and mediate anti-tumor immunity and immunotherapy responses. While single-cell T and B cell receptor sequencing have revealed insights into the clonal dynamics of adaptive immunity, the lack of analogous tools has precluded similar analysis of innate immune cells. Here, we describe a method leveraging somatic mitochondrial DNA (mtDNA) mutations to reconstruct clonal lineage relationships between cells in native human tissues. By jointly profiling single-cell chromatin accessibility and mtDNA variants, we resolve clonal dynamics of 218,715 cells from matched tumors, tissues, and blood from patients with lung and ovarian cancers. Clonal tracing reveals that TME-resident myeloid subsets, including macrophages and type 3 dendritic cells (DC3), are clonally related to circulating and tissue-infiltrating monocytes. We further identify distinct DC-biased and macrophage-biased clones, whose circulating monocyte precursors exhibit distinct epigenetic profiles, suggesting intratumoral myeloid differentiation fate may be peripherally programmed before TME infiltration.

## In brief

Liu et al. develop Mitotrek, a method that uses single-cell multi-omics with mitochondrial DNA clonal tracing to map the clonal origins of innate immune cells in human tumors. They reveal that tumor myeloid populations arise from circulating monocytes with pre-existing epigenetic fate biases, suggesting that tumor immunity is shaped before tumor infiltration.

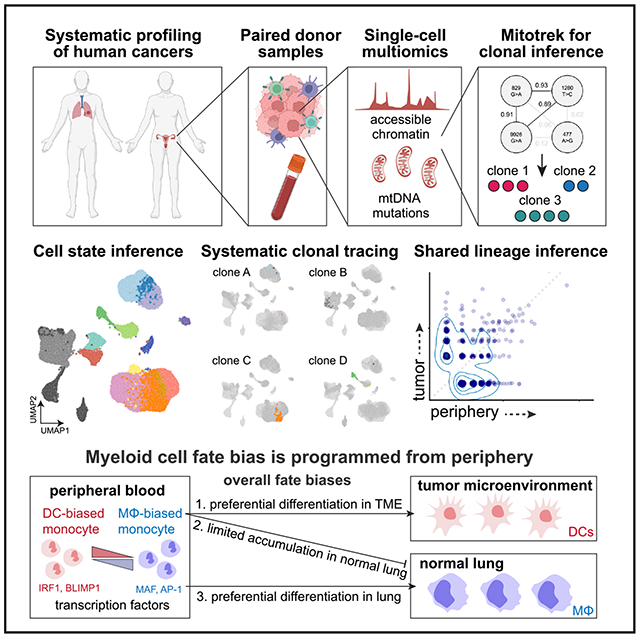

## INTRODUCTION

The tumor microenvironment (TME) is a dynamic ecosystem that maintains a close connection to the peripheral immune system through the bloodstream. Circulating immune cells continually infiltrate the TME, where their capacity to differentiate and expand shapes anti-tumor immunity and therapy response. Lineage tracing methods such as paired single-cell RNA and TCR sequencing (scRNA/TCR-seq) have advanced understanding of the expansion potential, tissue distribution, and clonal dynamics of immune populations in patients.^1,2^ For example, we and others have applied scRNA/TCR-seq to show that peripherally expanded, tumor-specific CD8^+^ T cell clones replace dysfunctional exhausted T cells in the tumor, which is essential to immune checkpoint blockade response.^3–5^ However, similar lineage-tracing approaches have not been feasible for non-adaptive immune populations due to the absence of somatic recombination events during their development.

Innate immune cells, including myeloid cells, are among the most abundant immune cell types in the TME^6^ and are ontogenetically diverse, consisting of both tissue-resident and circulating bone marrow-derived compartments.^7^ Although phenotypic and quantitative maps have delineated their diversity across tissues and cancer types,^8–11^ and there is growing recognition that ontogeny plays a key role in shaping their function within the TME,^12,13^ the inability to connect cellular phenotype and lineage within native human tissues leaves several open questions. First, do innate immune cells have an intrinsic tissue site preference for infiltration, differentiation, or expansion? Second, do they face clonally selective pressures in the TME, analogous to those experienced by T and B cells? Finally, what specific lineage relationships connect the diverse innate immune constituents within the TME, and what are their ontogenetic origins?

Somatic mtDNA variants have been established as endogenous lineage markers, as the high mutation rate of mtDNA relative to nuclear DNA drives the accumulation of lineage-distinguishing mutations over time.^14,15^ Recent advancements in single-cell sequencing have enabled the detection of mtDNA mutations^16–19^ allowing comprehensive, simultaneous lineage and epigenetic profiling of thousands of clonotypes across cell types, tissue sites, and cancer types. Therefore, to address these fundamental questions regarding innate immune cell clonality and ontogeny, we performed mitochondrial single-cell assay for transposase accessible chromatin by sequencing (mtscA-TAC-seq) on 218,715 cells from tumor, non-involved lung tissue (NILT), and/or peripheral blood mononuclear cells (PBMCs) from patients with lung or ovarian cancer to provide a clonally resolved view of the human innate immune response to cancer.

## RESULTS

### Development of Mitotrek to recover single-cell clonal relationships

**Figure 1.**
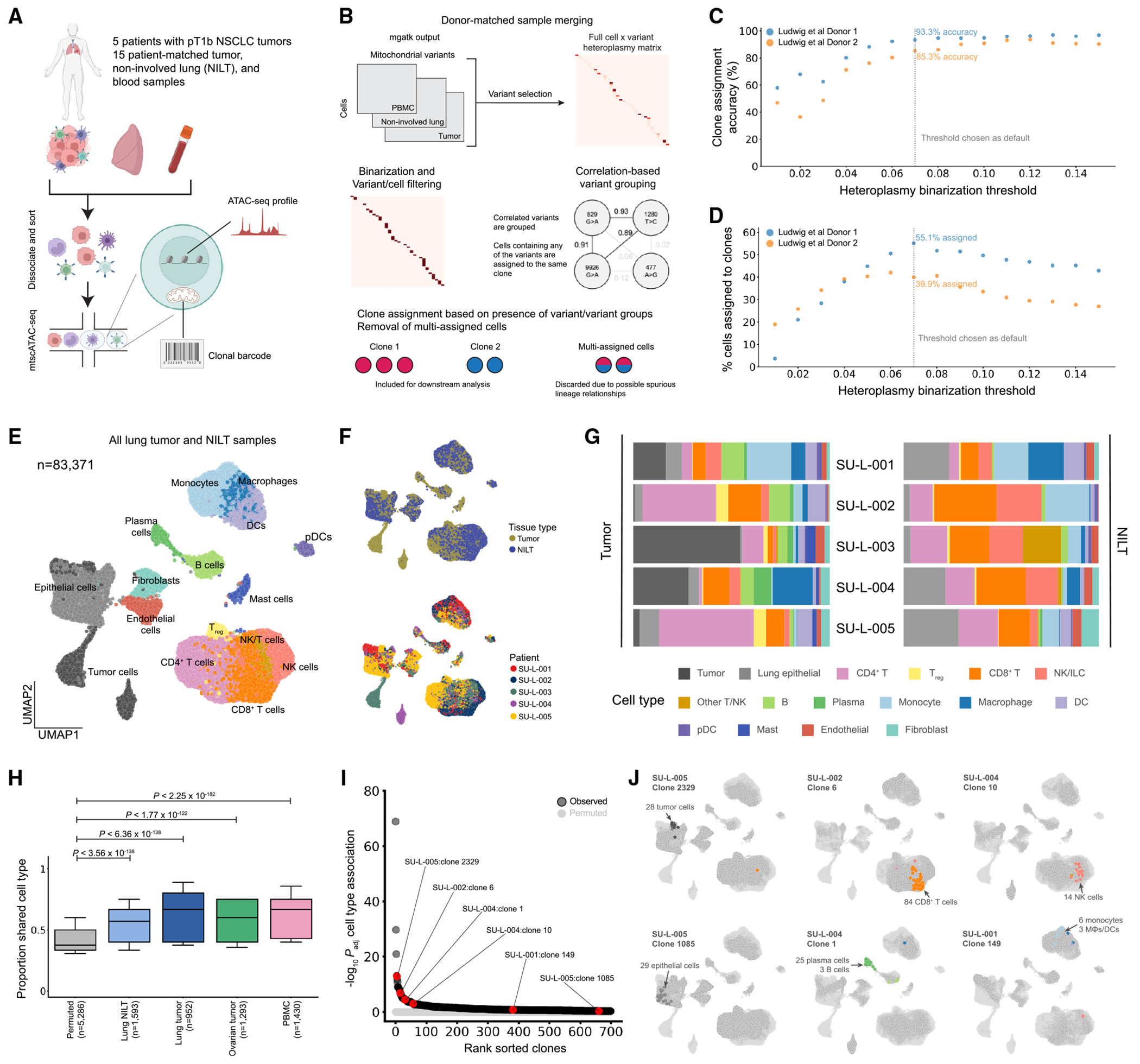
Creating a lineage-embedded atlas of NSCLC from mitochondrial DNA with Mitotrek (A) Schematic of simultaneous single-cell epigenetic profiling and clone tracing in patients with early-stage NSCLC. The chromatin accessibility profile and mitochondrial mutations are recovered from each cell. (B) Schematic of clone calling from mitochondrial variants using Mitotrek. (C) Benchmarking Mitotrek on gold-standard HSPC colony data. (D) Same as (C) but for a second HSPC donor. (E) Uniform manifold approximation and projection (UMAP) of 83,371 cells in lung tumor and non-involved lung tissue (NILT). (F) UMAP of cells colored by tissue type (top) and patient identity (bottom). (G) Normalized bar plot showing cell type composition for each patient, partitioned by tumor and non-involved lung. (H) Distribution of the proportion of cells within each clone (≥3 cells) that share the most common cell type for that clone compared to permuted data from the same samples. *N* = number of clones, Kruskal-Wallis test. Boxplots: center line, median; box limits, first and third quartiles; whiskers, 1.5× interquartile range. (I) Clone associations with cell type. *p* values represent the Benjamini-Hochberg adjusted Kruskal-Wallis test against overall cell type proportions for clones with at least five cells. Clones shown in (J) are highlighted in red. (J) Representative clones capturing clonal expansion (tumor, CD8+ T, NK, and epithelial) or differentiation (B/plasma and monocyte/macrophage) events in tumor and NILT. See also Figures S1 and S2; Tables S1 and S2.

To establish a lineage tracing method applicable to donor-matched solid tissue and blood samples and to clonally link immune cells across tissue sites, we applied mtscATAC-seq to profile five patients with early-stage non-small cell lung cancer (NSCLC) who had undergone therapeutic lobectomy, spanning adenocarcinoma (SU-L-002, SU-L-004, SU-L-005; treatment-naïve), squamous cell carcinoma (SU-L-001; post-neoadjuvant chemotherapy), and neuroendocrine (SU-L-003) histologies (Figures 1A and S1A; Table S1; methods). To investigate whether distinct TMEs influence immune clonal architecture, we complemented the NSCLC dataset, characterized by high mutational burden and strong immune infiltration,^20,21^ with mtscATAC-seq data from five ovarian cancer patients, which typically exhibit a more immunosuppressive TME^22^ (Figure S1B; Table S1). Fresh samples were dissociated and used to generate single-cell ATAC and mtDNA genome-sequencing libraries, enabling simultaneous recovery of chromatin accessibility profiles and mtDNA genotypes from individual cells.

In the lung cancer cohort, we obtained mtscATAC-seq profiles from 83,371 immune, malignant, and stromal cells from tumor and NILT, and 41,587 profiles from peripheral blood. In the ovarian cancer cohort, we profiled 52,154 immune, malignant, and stromal cells from ovarian tumors and 41,603 PBMCs (Table S2; Figure S1C). For one patient (SU-O-002), we additionally generated paired scRNA/TCR-seq data from 6,982 cells spanning peripheral blood and tumor, with TCR-mtDNA correspondence supporting clonal barcoding of immune cells in this dataset^16^ (Figure S1D; methods). We recovered 10,584 high-confidence somatic mtDNA mutations present at low pseudobulk frequencies (<1%) as determined by *mgatk*^17^ (Figure S1E), a computational pipeline to process mitochondrial reads and generate heteroplasmy estimates from mtscATAC-seq data. Variants with >1% pseudobulk frequencies, accounting for 0.76% of all variants detected, were broadly distributed across cell types and tissue sites, likely representing early-developmental or zygotic mutations^23^ that are not informative for resolving recent immune lineage relationships (Figure S1F), and were therefore excluded from downstream analysis.

Analysis of heteroplasmy distributions revealed that mtDNA variants detected at high allele frequencies in solid tissue cells were more frequently shared across cells, including from other lineages, than in matching PBMC samples, likely due to both technical artifacts (e.g., uptake of ambient mtDNA during single-cell capture^24^) and biological phenomena (e.g., horizontal mitochondrial transfer events^25^) that are unlikely to reflect *bona fide* clonal relationships and may be unique to solid tumor profiling^18,26,27^ (Figures S1G–S1L). To mitigate such spurious lineage inferences, we developed Mitotrek, a computational framework that assigns cells to high-confidence clones by prioritizing clonal accuracy over sensitivity (Figure 1B; methods). Briefly, Mitotrek: (1) reformats cell-by-variant heteroplasmy data into a binary ‘‘positive-unlabeled’’ matrix using empirically defined thresholds to account for measurement noise from genetic drift^28^ and variable sequencing depth, (2) discards variants detected in >20% of cells within a sample, which are likely to generate artifactual linkages, and (3) excludes cells assigned to multiple clones to avoid contamination by non-informative variants of technical or biological origin. Subclonal structure was not explored due to the difficulty in establishing true clonal hierarchy, and potential subclonal relationships were collapsed to single clones by merging highly correlated variants. Benchmarking Mitotrek on full-length scRNA-seq data from single-colony hematopoietic stem and progenitor cells (HSPCs) with known clonal identities^16^ yielded assignment accuracies of 91.3% and 85.3% for two independent donors (Figures 1C, 1D, S1M, and S1N), establishing Mitotrek as a high-accuracy framework for resolving clonal relationships in solid human tissues.

### Constructing multi-modal atlases of NSCLC and ovarian cancer

We next constructed a single-cell epigenetic atlas of NSCLC by iteratively clustering scATAC-seq profiles from tumor and NILT samples, identifying 53 cell clusters spanning malignant tumor, epithelial, stromal, T cell, NK/ILC, myeloid, B cell, and plasma cell populations (Figures 1E; Figure S2A). All non-malignant cell-type annotations contained cells from multiple donors, consistent with interpatient heterogeneity,^29^ and cell-type assignments aligned with surface markers used for enrichment (Figures 1F and S2B). Cell-type distributions in tumor and NILT samples matched previous transcriptomic and proteomic datasets^8,30^ (Figure 1G). In patient-matched PBMCs, we recovered all major immune populations with the expected absence of tissue-resident myeloid subsets, and additionally identified circulating HSPCs (Figures S2C–S2F), enabling unbiased downstream lineage analysis.

Applying Mitotrek to the NSCLC dataset, we recovered 5,146 clones comprising 29,010 cells (23.2% of cells passing scATAC-seq QC filters), of which 3,190 clones (62%) contained ≥3 cells and 573 (11%) contained ≥10 cells. The number of clones recovered per patient (440–2,146) was highly correlated with cell recovery (R = 0.90; Figure S2G), and clone assignment rates were consistent across cell types and tissue sites, ranging from 15% to 26% in tumor/NILT and 20%–32% in PBMC (Figures S2H and S2I), with the fraction of multi-assigned cells decreasing with clone size (Figures S2J and S2K). Globally, cells within the same clone were significantly more likely to share a cell type than randomly grouped cells across all tissues (Figure 1H), reflecting lineage fidelity and supporting the validity of Mitotrek-assigned clones. A clone-level cell type association analysis identified 226 clones (20% of clones with ≥5 cells) whose compositions significantly deviated from the background distribution (Padj < 0.05; Figure 1I), capturing key expansion and differentiation events across immune, stromal, and epithelial compartments (Figure 1J).

Iterative clustering of ovarian tumor cells identified 40 clusters across the same broad cell type categories (Figures S3A–S3E). Mitotrek recovered 4,560 clones comprising 20,713 cells, of which 2,389 clones (53%) contained ≥3 cells and 287 clones (6%) contained ≥10 cells. The number of clones recovered per patient ranged from 61 to 1.487 (Figure S3F). Consistent with our observations in the NSCLC dataset, clone counts were strongly correlated with cell recovery per patient (R = 0.93), and clone assignment rates were uniform across cell types and tissue sites (Figures S3F–S3J). Together, the lineage-embedded NSCLC and ovarian tumor datasets provide a robust framework for dissecting lineage dynamics across distinct TMEs.

### Immune clonal landscapes across distinct tissue sites reveal a diverse clonal repertoire of tissue-infiltrating myeloid cells

**Figure 2.**
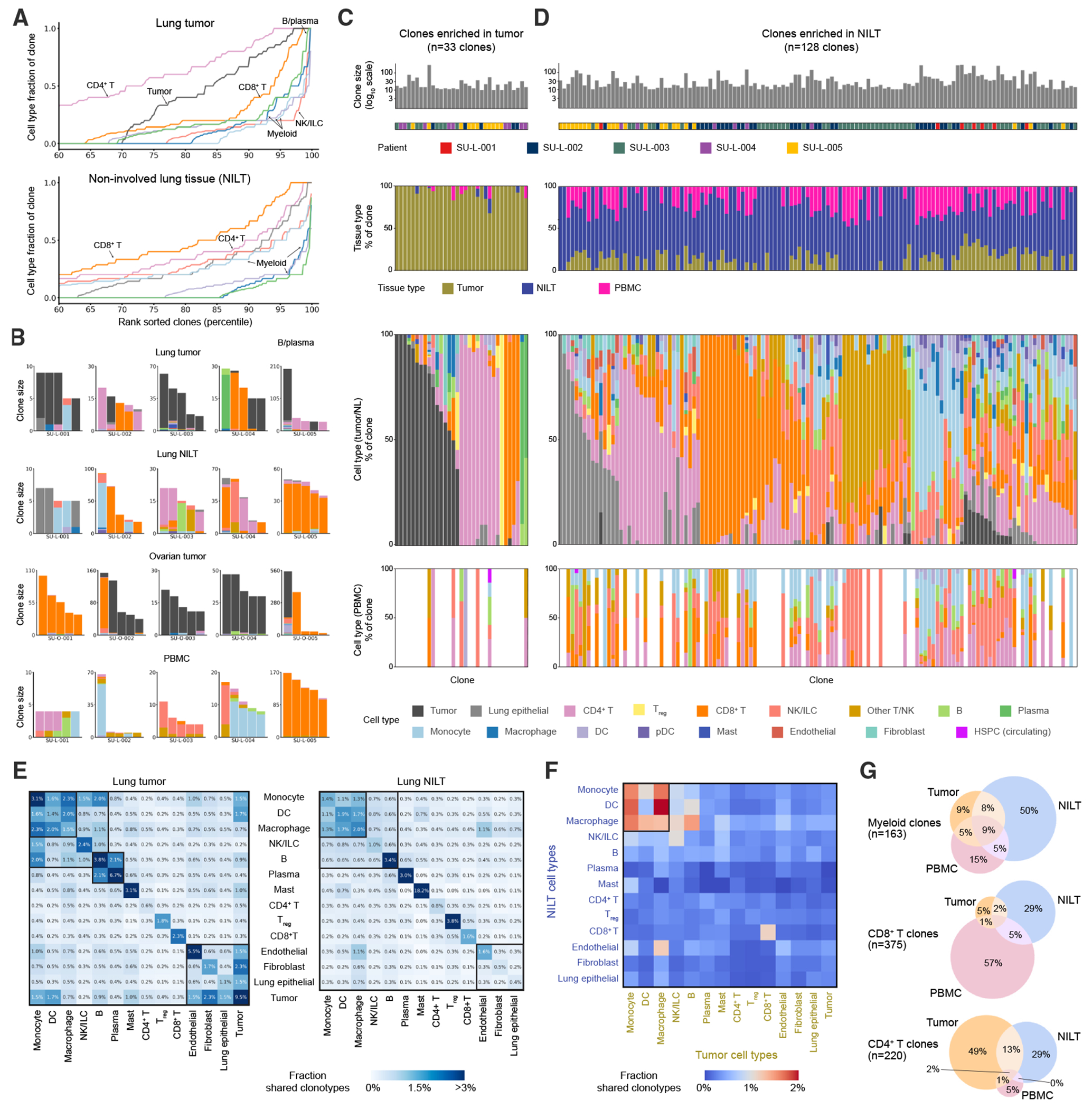
Cross-tissue clonal landscape of tumor-infiltrating immune cells (A) Cumulative fractions of clones stratified by cell type for cells from lung tumor (top) and NILT (bottom). Clones with ≥5 cells are considered for this analysis. (B) Cell types of single cells belonging to the same clone. The top five most abundant clones with the most common cell type >70% for each patient in the indicated sample are shown. Each bar is colored by cell types of single cells within the clone. (C) Clones enriched in tumor, as determined by *p* value < 0.05 from Benjamini-Hochberg adjusted Fisher’s exact test against overall tissue site distribution for clones with at least 10 cells in tumor and NILT. Each column represents a unique clone. (D) Same as in (C) but for the NILT. (E) Heatmaps showing the fraction of all cell pairs belonging to the same clone and consisting of two cell types within lung tumor (left) and NILT (right). Pairs were restricted to cells from the same donor. (F) Heatmap showing the fraction of all cell pairs belonging to the same clone and consisting of a lung tumor cell type and a NILT cell type. (G) Comparison and overlap of clones (≥3 cells for the indicated cell type) for myeloid, CD8+ T, and CD4+ T. Myeloid consists of monocyte, macrophage, and DC. n = number of clones considered for each cell type. See also Figures S3–S7

As mtscATAC-seq enables a measure of clonal diversity across all cell states, we assessed the relative clone sizes of both immune and tumor cells from NSCLC and ovarian cancer samples. We quantified the distribution of clone fractions by cell type, reasoning that high per-clone fractions reflect cell type-specific clonal expansion events and noting that mitochondrial lineage tracing is particularly adept at marking clonal expansions^31^ (Figures 2A and S3K). Overall, tumor cells and adaptive immune cells, including T cells, B cells, and plasma cells, comprised the largest clones observed in both lung and ovarian tumors (Figures 2B and S4A). Among clones composed of ≥80% of a single cell type (≥3 cells per clone), lung tumors were dominated by tumor cells, CD8^+^ T cells, CD4^+^ T cells, and B/plasma cells, whereas ovarian tumors had notably lower representation of CD4^+^ T cells, suggesting that clonally expanded CD4^+^ T cells are more prominent in the lung TME (Figures S4B–S4F). This inference was corroborated by matched ovarian tumor scRNA/TCR-seq, which showed markedly reduced clone sizes for CD4^+^ compared to CD8^+^ T cells (mean 7.6 vs. 25.4 cells per TCR clone; Student’s *t* test *p* = 0.029; Figure S4G).

Myeloid populations, including monocytes, macrophages, and DCs, exhibited smaller clone fractions, compared to adaptive immune cells. Among clones with a substantial number (≥5) of myeloid cells detected in lung tumors, the mean myeloid fraction was 58.5%. In contrast, clones with a substantial number of CD4^+^ T cells, CD8^+^ T cells, and B/plasma cells exhibited higher mean fractions of 78.7%, 78.3%, and 62.1% of the corresponding cell types (Figure S4H). Similar patterns were observed in ovarian tumors, except for the lower level of clonal expansion of CD4^+^ T cells (Figures S4I–S4K). These data provide direct evidence that sustained clonal expansion of human myeloid cells following bone marrow egress is limited, compared to lymphoid cell types, which clonally expand following antigen recognition. Notably, we observed oligoclonal NK cell expansions in one ovarian tumor (SU-O-005), consistent with adaptive-like NK responses reported in other contexts^26,32–35^ (Figures S5A–S5D).

We next compared the clonal landscape of lung tumors to the paired normal lung tissue environment. Overall, clone sizes were correlated across tissue types, with the strongest concordance observed between NILT and blood and weaker correlation between blood and tumor (Figure S6A). We classified clones with ≥10 cells based on their enrichment in tumor versus NILT (Figure S6B). Tumor-enriched clones were dominated by tumor cells as well as CD4^+^ T, CD8^+^ T, and B/plasma cells, with the adaptive immune clones largely restricted to the tumor site (Figure 2C). In contrast, NILT-enriched clones included a broader range of cell types with higher representation across tissue sites (Figure 2D), including clones predominantly composed of invariant NK/T cells and monocytes that were minimally observed in tumor-enriched clones. NILT-enriched clones also showed greater cellular heterogeneity than tumor-enriched clones (Figure S6C), consistent with reduced clonal expansion in the normal lung tissue environment.

Among the 186 clones that were not preferentially enriched in either tumor or NILT, most were dominated by CD4^+^ or CD8^+^ T cells (Figure S6D) and were detected across tumor, NILT, and peripheral blood, consistent with previous reports demonstrating concordance between peripheral and intratumoral T cell clone sizes.^3,4^ In contrast, B cell and plasma cell-dominated clones were restricted to either tumor or NILT, suggesting that B and plasma cell expansion is locally confined. A complementary analysis of PBMC-enriched clones identified 14 clones dominated by CD8^+^ T and NK cells, the majority of which were also detected at lower frequencies in tumor and NILT, indicating their capacity for tissue infiltration (Figure S6E). Together, these results provide a comprehensive view of immune clonal composition across the human TME and periphery.

### Inter-cell type clonal relationships reveal broad tissue distribution of bone marrow-derived myeloid cells

Next, we systematically quantified lineage relationships and differentiation patterns between cell types within and across tissue sites. We aggregated all clonotypes for each pair of cell types (including self-self pairs) and measured the fraction of clones shared across distinct cell-pair combinations, reasoning that two cell types were more related if they more frequently shared clonotypes (Figures 2E and S7A; methods). Across all tissue samples, we identified three broad patterns: (1) innate immune cells (monocytes, macrophages, DCs, and NK cells) consistently exhibited high intra-group clone sharing, consistent with recent hematopoietic output without substantial clonal bottlenecks prior to tissue infiltration, (2) adaptive immune cells and tumor cells showed high intra-cell type clone sharing, reflecting histories of clonal selection and expansion, and (3) stromal cells (endothelial cells, fibroblasts, and non-tumor epithelial cells) displayed intermediate levels, consistent with their distant embryonic origins relative to HSPC-derived immune cells. Analogous patterns were observed in PBMCs (Figure S7B). Notable exceptions included non-regulatory CD4^+^ T cells, which exhibited minimal intra-cell type clone sharing despite the expansion of select clones, indicating broader clonal diversity, and endothelial cells, which displayed particularly high intra-cell type sharing in lung and ovarian tumors but not NILT, consistent with the angiogenic processes indispensable for tumor formation.

We next quantified lineage relationships between cell types across patient-matched lung tumors, NILT, and peripheral blood. Among adaptive immune cells, only CD8^+^ T cells consistently exhibited high clone sharing across tissue sites, suggesting that peripheral expansion during the anti-tumor response is unique to this population (Figures 2F and S7C). In contrast, B and plasma cells across tissues were clonally distinct, supporting the notion that B cell-mediated immunity is locally orchestrated, as has been recently noted in the context of infection.^36^ Among myeloid cells, we observed the greatest degree of clone sharing across tissue sites, suggesting that these cells readily infiltrate and differentiate in tissue following bone marrow myelopoiesis and egress. Indeed, myeloid-dominated clones spanning multiple tissue sites were the most frequently detected in the NSCLC dataset (27% myeloid clones compared to 8% CD8^+^ T cell clones and 16% CD4^+^ T cell clones; Figure 2G). Together, these findings emphasize the distinct clonal dynamics of adaptive and innate immune cells in anti-tumor immunity, highlighting the systemic nature of CD8^+^ T cell responses in contrast to the locally restricted nature of B cell responses, and revealing the extensive tissue infiltration and differentiation capacity of bone marrow-derived myeloid cells.

### Intratumoral DC3s are epigenetically and clonally linked to circulating monocytes

**Figure 3.**
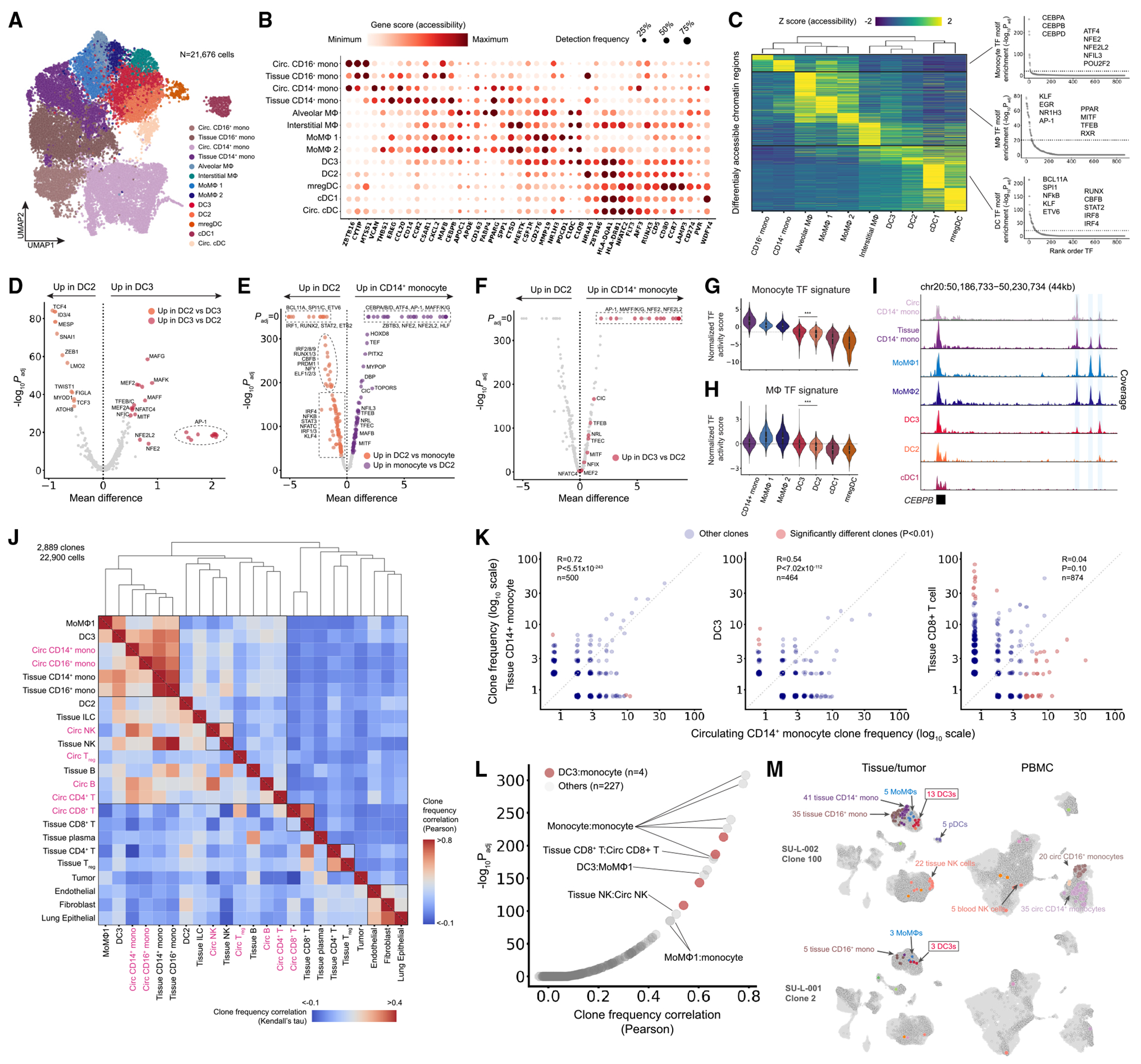
Intratumoral DC3s are epigenetically and clonally related to monocytes (A) UMAP of myeloid cells from PBMC, lung tumor, and NILT samples of patients with lung adenocarcinoma (LUAD). (B) Column-scaled gene accessibility scores and detection frequencies for the indicated genes. (C) (Left) Heatmap showing marker peaks for all myeloid cell types. The color indicates column-scaled, mean-adjusted number of reads detected in each peak. (Right) Representative TF motifs that are significantly enriched for monocyte, macrophage, and DC. (D) Differentially active TF motifs between DC2 and DC3. *p* values are calculated using the Benjamini-Hochberg adjusted Kruskal-Wallis test. (E) Same as (D) but comparing DC2 and CD14+ monocytes. (F) Same as (D) but highlighting DC3-up TF motifs with increased accessibility. (G and H) Average chromVAR motif deviation scores for monocyte and macrophage TF motifs highlighted in (C). Kruskal-Wallis test. (I) Chromatin accessibility tracks for the CEBPB locus for the indicated cell types. (J) Cell type-cell type clone frequency correlation across clones (≥5 cells across all samples). Color denotes correlation value, computed using Pearson’s ρ (upper half), and Kendall’s τ (bottom half). Text labels of circulating PBMC cell types are colored pink. (K) Scatterplots comparing clone frequencies of circulating CD14+ monocytes with those of tissue CD14+ monocytes (left), DC3 (middle), and tissue CD8+ T cells (right). (L) 231 distinct cell type pairs ordered by clone frequency correlation. DC3-monocyte interactions are highlighted in red. (M) Representative clones consisting of DC3 and monocytes. For each clone, cell types with at least two cells are highlighted. See also Figures S8–S10.

It has been reported that specific subpopulations of circulating myeloid cells give rise to tumor-resident mononuclear phagocyte (MNP) populations that critically shape the TME.^13,37,38^ In particular, human DC3s have been identified as a CD1c^+^ dendritic cell subset distinct from cDC2s and enriched in NSCLC tumors, and have been suggested to uniquely prime tissue-resident CD8^+^ T cells.^30,39–42^ However, the epigenetic and ontogenetic relationships between human DC3s and other MNPs remain undefined. Given the broad tissue distribution of bone marrow-derived myeloid cells, we sought to understand the differentiation trajectories of circulating myeloid populations and clonally link them to those in the TME. To achieve higher granularity for myeloid cell type annotation, we iteratively re-clustered 21,676 myeloid cells from lung tumor, NILT, and peripheral blood in the NSCLC cohort into 13 distinct clusters, including classical (CD14^+^) and non-classical (CD16^+^) monocytes, alveolar macrophages (AMΦ), interstitial macrophages (IMΦ), two monocyte-derived macrophage populations (MoMΦ1 and MoMΦ2), and DC subsets including cDC1, cDC2, mature DC enriched in regulatory molecules (mregDC),^9,43,44^ and DC3s^30^ (Figures 3A, 3B, and S8A–S8C; methods). Annotations were validated using gene signatures derived from joint single-cell transcriptomic and proteomic profiling of human lung myeloid cells from 35 NSCLC tumors and 29 patient-matched NILTs^30^ (Figures S8D and S8E), and were consistent with previous characterizations of human lung tumors and NILTs.

Despite extensive transcriptomic and proteomic characterization of lung myeloid cells, the corresponding epigenetic landscape remains understudied. We used ArchR^45^ to identify differentially accessible regions (DARs) specific to each myeloid cell subtype. Although subtypes exhibited distinct epigenetic profiles, hierarchical clustering grouped them into three broader categories reflecting their myeloid lineage, with IMΦ cells excluded from downstream analyses due to their rarity across donors (96% originated from a single donor; Figure 3C). Transcription factor (TF) motif enrichment analysis of subtype-specific DARs recapitulated established lineage-defining programs, including CEBP motifs in monocytes,^46–52^ AP-1 and metabolic receptor family (RXR, PPAR, and LXRα) motifs in macrophages,^48–51,53–60^ and canonical DC TFs (BCL11A, IRF4/8, RUNX, CBFB, and NF-κB) in DCs^48–51,61–63^ (Figures S8F and S8G).

Unlike cDC2 DARs, DC3-enriched DARs exhibited significantly greater accessibility in monocytes and macrophages (Figures S9A and S9B), and CD14^+^ monocyte DARs were more accessible in DC3s than in other DC subtypes (Figure S9C), suggesting that DC3s retain monocytic epigenetic features. TF motif analysis of DC3 DARs revealed increased accessibility for motifs associated with monocyte and macrophage TF families, including AP-1, MAF, and MiT-TFE (Figures 3D–3F). Using chromVAR, we inferred per-cell TF activity and observed concurrent activation of monocyte-, macrophage-, and DC-associated TF programs in DC3s (Figures 3G–3I and S9D), with CD14^+^ but not CD16^+^ monocytes showing anti-correlated accessibility of monocyte- versus DC-associated motifs, suggestive of transitional states during monocyte-to-DC differentiation (Figures S9E and S9F). Increased RNA expression of AP-1 family members in DC3s from published scRNA-seq data^30^ further supported these observations (Figure S9G). Collectively, DC3s harbor monocytic epigenetic features indicative of a shared ontogeny distinct from classical DCs.

To reconstruct lineage relationships between DC3s and other immune subsets independent of epigenetic profiles, we analyzed clonal frequency correlations across immune subtypes, reasoning that ontogenetically related populations are also clonally related. As expected, lineage proximity between circulating and tissue-infiltrating monocytes was recapitulated (Figure 3J). Strikingly, DC3s clustered closely with monocytes and MoMΦs (Figures 3K and 3L), whereas cDC2s were less clonally related to monocytes (Figure S9H). For example, a representative clone defined by the 3068G>A mtDNA mutation showed 35 CD14^+^ monocytes in the peripheral blood, 41 CD14^+^ monocytes in the tissue, and 13 DC3s in the tumor and NILT, likely reflecting continuous infiltration and differentiation (Figure 3M). These findings were recapitulated in ovarian tumors, where intratumoral DC3s showed similar monocyte/macrophage TF-motif enrichment, concordant chromVAR activity, and clonal proximity to circulating monocytes (Figures S10A–S10J), suggesting the monocyte-DC3 connection is tumor-agnostic.

### Biased monocyte differentiation fates peripherally reprogram the tumor myeloid compartment

**Figure 4.**
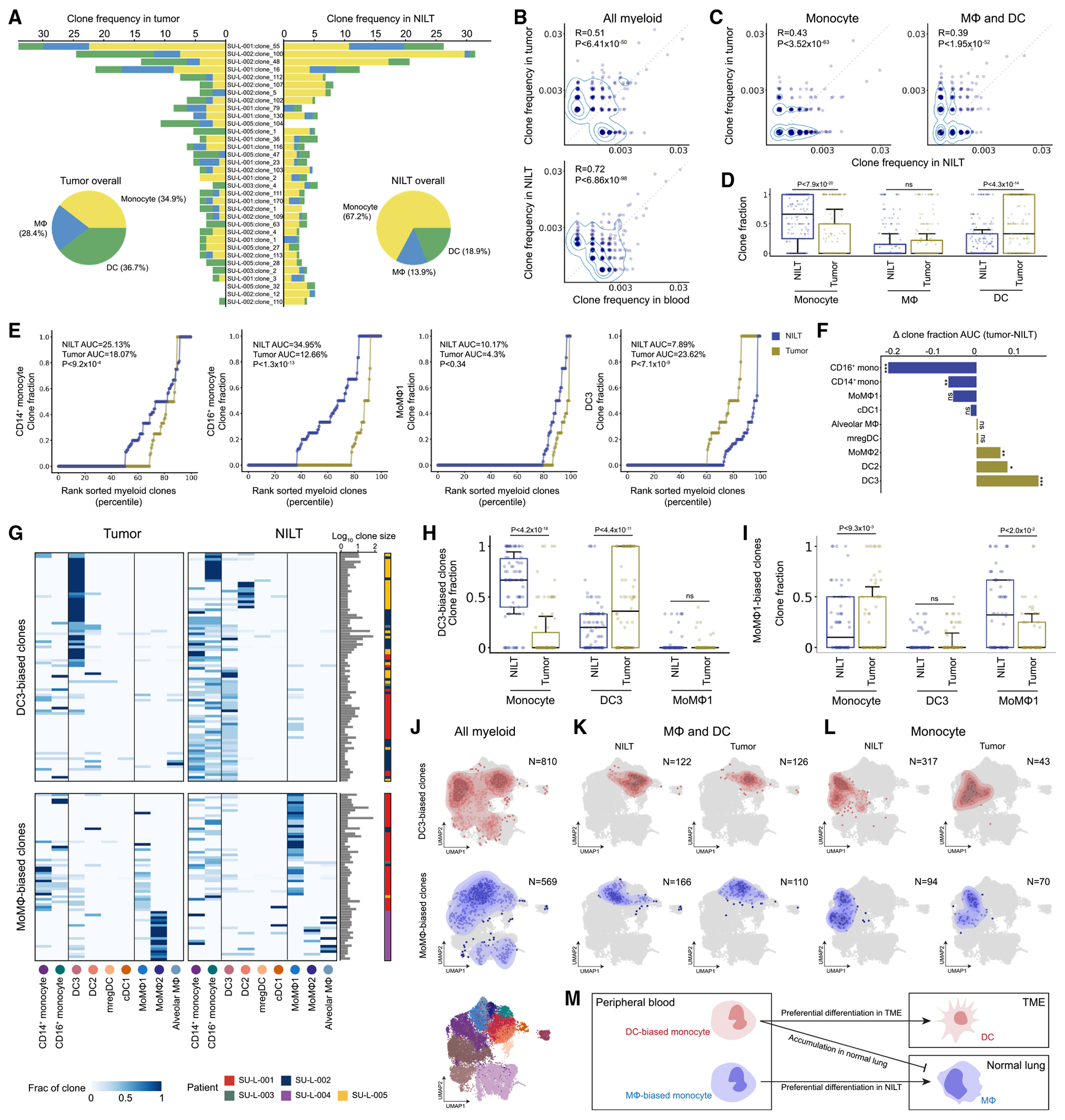
Divergent clonal myeloid differentiation fates in human tissues (A) Monocyte, macrophage, and DC proportions of largest myeloid clones (≥10 cells), split by tumor and NILT. (B) Scatterplots comparing clone frequencies of circulating myeloid cells with those in tumor (top) and NILT (bottom) myeloid cells. R and *p* value from Spearman’s correlation. (C) Scatterplots comparing clone frequencies of monocytes (left) and macrophage/DC (right) between NILT and tumor. R and *p* value from Spearman’s correlation. (D) Distribution of the cell type fraction within each myeloid clone (≥5 cells), split by tissue site. *p* values are from a Wald statistic from binomial generalized linear model (GLM) with a logit link. Boxplots: center line, median; box limits, first and third quartiles; whiskers, 1.5× interquartile range. (E) Cumulative fraction of clone sizes for the indicated myeloid cell types, split by tissue site. AUC corresponds to the overall clone size for the indicated cell type and tissue site. (F) Summary of AUC differences between tumor and NILT for all myeloid cell types. A positive value indicates the cell type has larger clone sizes in lung tumors compared to NILTs. Significance is determined by *p* values from (D) and (E). (G) Heatmaps showing cell type proportions split by tumor and NILT for DC-biased (top) and MΦ-biased (bottom) clones. Clone sizes correspond to the number of myeloid cells per clone. Only clones detected in both tissue sites or clones with at least three cells are used to identify cell-type biased clones (H) Distribution of cell type fraction within DC3-biased clones. (I) Same as in (H) but for MoMΦ1-biased clones. (J) Contour plots, representing cell density of DC3-biased clones (top row, red) and MoMΦ-biased clones (bottom row, blue), projected onto the UMAP. (K) Same as (J) but highlighting tissue-derived monocytes. (L) Same as (J) but highlighting MΦ/DC.(M) Schematic illustrating divergent clonal differentiation fate for myeloid cells. See also Figure S11 and Table S3

While circulating monocytes have the capacity to differentiate into either macrophages or DCs as they infiltrate inflamed tissue,^53,57,63–65^ it is not clear whether differentiation fates are entirely dictated by the tissue environment or there is cell-intrinsic bias in the context of human cancer. To address this question, we next asked whether myeloid clones displayed tissue-specific differentiation preferences by comparing their frequencies across lung tumor, NILT, and peripheral blood (Figures 4A, 4B, S11A, and S11B). Overall, clone frequencies were highly correlated across sites, and the largest clones in tumors and NILT were also detected in blood, indicating infiltration from circulation without tissue site-specific expansion biases. However, within clones, monocytes were proportionally more abundant in NILT, whereas differentiated macrophages and DCs were enriched in tumors (Figures 4C, 4D, and S11C). These observations are consistent with the notion that TME promotes monocyte differentiation, likely driven by elevated inflammatory cues.^65^

To identify the specific myeloid subtypes that monocytes preferentially differentiate into within the TME, we analyzed clone compositions by subtype (Figures 4E, 4F, and S11D). Both CD14^+^ and CD16^+^ monocytes were less represented in tumors compared to NILTs, while SPP1^+^ MoMΦ2 and DC3s were preferentially enriched in tumors. Notably, MoMΦ1 was more abundant in NILTs. If differentiation were purely driven by the tissue environment, the same clone would display different myeloid subtype compositions between tumor and NILT. To test this hypothesis, we clustered clone-level subtype compositions stratified by tissue site (Figure 4G). Interestingly, we observed clone-intrinsic lineage biases, with clones consistently favoring differentiation toward either DCs or macrophages, independent of tissue site. DC-biased clones were enriched for DC3s in tumors and consisted primarily of monocytes in NILTs, with minimal macrophage representation across sites (Figure 4H). In contrast, macrophage-biased clones exhibited distinct behaviors depending on the MoMΦ subtype: MoMΦ1-biased clones were depleted of monocytes and enriched for MoMΦ1 cells in NILTs (Figure 4I), whereas MoMΦ2-biased clones almost exclusively consisted of MoMΦ2 cells in tumors. These findings suggest that monocytes possess intrinsic biases in both differentiation fate and site preference.

We next grouped myeloid cells from DC-biased and macrophage-biased clones to compare their epigenetic profiles. Across tissue sites, cells from the two groups clustered distinctly, even within the same myeloid subtype such as CD14^+^ monocytes in both tissue and periphery (Figures 4J–4M). Remarkably, circulating CD14^+^ monocytes from the two groups were epigenetically distinct even prior to tissue infiltration, with monocytes within each group more similar to one another than to those in the other group (Figures S11E–S11H). TF motif analysis revealed that DC-biased monocytes were enriched for proinflammatory programs (NF-κB, IRF, STAT2, and BLIMP-1), whereas macrophage-biased monocytes were enriched for immunosuppressive programs (MAF family, NRF2, and RUNX1/2)^66–71^ (Figure S11I). Upon tumor infiltration, IRF and BLIMP-1 motifs remained differentially accessible in DC-biased monocytes, with ID3/ID4 additionally becoming selectively accessible, supporting their anti-tumor potential^72^ (Figure S11J).

Corroborating these motif-level inferences, gene body accessibility analysis identified *IRF1, IRF3*, and *BLIMP-1* as differentially accessible in DC-biased monocytes and *MAF* (encoding c-MAF, a key driver of macrophage immunosuppressive phenotypes^73–75^) in macrophage-biased monocytes, with cross-tissue patterns suggesting stage-specific roles before and after tissue infiltration (Figures S11K and S11L). To further support these inferences, we reanalyzed scRNA-seq data from a comprehensive atlas of myeloid cells in human lung tumors^8,30^ and performed pseudotime inference to identify fate-biased peripheral monocytes to both the macrophage and DC3 lineages (Figures S11M–S11P; methods). Differential expression analyses of peripheral CD14^+^ monocytes confirmed fate-based transcriptional programs, including up-regulation of *C1QA, C1QB*, and *C1QC* in macrophage-biased monocytes and *FCER1A* and *EREG* in DC3-biased monocytes^76^ (Figure S11Q; full list in Table S3). Taken together, these findings suggest that circulating monocytes exhibit intrinsic epigenetic biases that predispose them to differentiate into either DCs or macrophages within the TME, contributing to their distinct functional roles in shaping the anti-tumor innate immune response.

## DISCUSSION

Here, we performed mtscATAC-seq on 218,715 cells across 23 matched tumor, non-involved tissue, and peripheral blood samples from patients with lung and ovarian cancers to generate a clonally resolved map of the human innate immune response to cancer. To ensure the accuracy of clonal tracing in solid tissues, we developed Mitotrek, a tailored analysis framework that prioritizes clonal assignment fidelity over sensitivity or resolution. Our analyses revealed that innate immune cells in tumors are primarily derived from a diverse pool of circulating precursors without evidence of tissue-specific clonal expansion, in contrast to the antigen-driven expansion of adaptive immune cells. Epigenetic and clonal analyses further revealed that DC3s, but not cDC2s, exhibit monocytic regulatory features and are clonally related to monocytes in both circulation and tissue. Although a recent study in mice showed that DC3s arise from bone marrow progenitors shared with monocytes,^77^ the rarity of DC3s in peripheral blood, their abundance in tumor lesions, and the lack of clonal expansion of DC3s in our data collectively suggest that human DC3s in tumors differentiate directly from monocytes upon tissue infiltration.

A key finding of our work is that phenotypically diverse tumor-infiltrating myeloid cells were clonally related to distinct subsets of circulating monocytes, with clones consistently biased toward either DC or macrophage fates in both tumor and non-involved tissue. These biases were accompanied by distinct chromatin accessibility patterns detectable even in peripheral blood monocytes prior to tissue infiltration: macrophage bias was linked to AP-1 family factors, c-MAF, and NRF2, whereas DC bias was linked to IRF, STAT2, and NF-κB motifs,^78^ suggestive of priming by type I interferons, TNF-α, and IL-1 signaling.^79,80^ Consistent with this, a recent study identified a macrophage subset defined by *FOSL2* (an AP-1 factor) activity and derived from circulating monocytes as strongly associated with glioma malignancy,^38^ supporting our observation that AP-1-enriched monocytes are predisposed to macrophage differentiation within the TME. These findings suggest that the tumor myeloid compartment may be peripherally programmed, offering an alternative to the view that intratumoral monocyte differentiation is primarily driven by local environmental cues, and raise the possibility that tumors epigenetically pre-condition monocytes through systemic cytokine signaling^81^ before tissue infiltration.

### Limitations of this study

Several limitations of our study could be addressed in future work. First, our findings are based on a modest cohort of patients with lung and ovarian cancers; although we deeply profiled each patient and observed consistent patterns across patients and cancer types, validation in additional patient settings, including other tumor types, will be important to establish generalizability. Second, our approach does not capture the temporal dynamics of monocyte infiltration and differentiation. Longitudinal sampling or integration with additional progenitor populations would further establish the sequence of events that shape the tumor myeloid compartment. Lastly, our current approach excludes nuclear-derived mutations that could, in principle, contribute to resolving subclonal relationships but are poorly covered by our assay. Future technological advances may enable more reliable inference of subclonal architectures to corroborate the differentiation dynamics that we propose.

## RESOURCE AVAILABILITY

### Lead contact

Further information and requests for resources and reagents should be directed to and will be fulfilled by the lead contact, Ansuman T. Satpathy (satpathy@stanford.edu).

### Materials availability

This study did not generate new reagents.

## STAR★METHODS

### EXPERIMENTAL MODEL AND STUDY PARTICIPANT DETAILS

#### Human samples

Fresh ovarian tumors, lung tumors, NILT samples, and peripheral blood were collected at the time of surgery by Stanford Tissue Procurement Shared Resource facility with the appropriate written informed consent and institutional IRB approval. Summary statistics and patient history are available in Table S1. For the early-stage NSCLC dataset, exclusion criteria included previous systemic treatment or radiotherapy. For the ovarian cancer dataset, exclusion criteria included tumors of non-ovary or unknown origin. We note that one patient (SU-L-003) had a neuroendocrine tumor and was receiving olaparib (PARP inhibitor) at the time of surgery for concurrent tubo-ovarian carcinosarcoma, which was confirmed as an independent primary tumor by pathology studies (Figure S1A).

### METHOD DETAILS

#### Tissue processing

For each patient, we collected tumor resections, peripheral blood samples, and non-adjacent non-neoplastic tissue (confirmed by pathological analysis) from the same resected lobe. Because the immediately adjacent tissue often exhibits pathological alterations, we excluded these tissues from genomic profiling. All tumor and non-adjacent healthy tissues were procured following surgical resection. Samples were dissociated and viably cryopreserved for downstream library preparation and sequencing. In brief, solid tumor specimens on ice were minced to pieces of <1 mm3 and transferred to 5 mL of digestion medium containing DNase I (100 μg/mL) and collagenase P (2 mg/mL) in Advanced DMEM/F-12. Minced tissue was transferred into C-tubes for use in the gentleMACS Octo Dissociator system at 37°C at 20 rpm for 20 min. After digestion, the cell suspension was filtered through a 70-μm filter, which was washed with an additional 10 mL of DMEM/F-12. The sample was then centrifuged at 400 g at 4°C for 5 min. Any residual undigested tissue was further digested for an additional 20-min incubation with additional digestion medium. After centrifugation, the supernatant was discarded, and the pellet was resuspended in 500 μL of ACK red blood cell lysis buffer and incubated for 1 min on ice, followed by the addition of ice-cold PBS. The cell count and viability were determined by trypan blue staining by using a Countess II FL automated cell counter, before proceeding to cell-sorting.

#### Fluorescence-activated cell sorting (FACS)

Cells were classified into T cells (CD45^+^CD3^+^), other leukocytes (CD45^+^CD3^−^), and malignant or stromal cells (CD45^−^ CD3^−^). The antibodies used included anti-human CD45 conjugated to V500 (clone HI30, 560779, lot 7172744, BD Biosciences) and anti-human CD3 conjugated to fluorescein isothiocyanate (FITC) (clone OKT3, 11-0037-41, lot 2007722, Invitrogen), both diluted at 1:200. Live/dead staining was performed using propidium iodide (P3566, Invitrogen) at a final concentration of 2.5 μg/mL. Cell sorting was conducted on a BD FACSAria III cell sorter (BD Biosciences).

#### Preparation of mtscATAC-seq libraries

For the generation of mtscATAC-seq libraries, we adapted the 10× Genomics scATAC-seq platform NextGEM v1.1 kits. In brief, mtscATAC-seq was performed with modifications to the ‘‘Nuclei Isolation for Single Cell ATAC Sequencing’’ (CG000169 Rev D) user guide, where we fixed and permeabilized cells to retain mitochondria and mtDNA within their host cell by removing Tween 20 as part of the lysis buffer.^19^ For the library preparation we followed the ‘‘Chromium Next GEM Single Cell ATAC Reagent Kits v1.1’’ (CG000209 Rev F), user guide with only minor modifications as described and highlighted below and otherwise refer the reader to the original and highly detailed workflow by 10× Genomics.^19^

#### Sequencing and upstream processing of mtscATAC-seq data

All libraries were sequenced on an Illumina Novaseq 6000 device using a 10 × 16 × 151×151 read configuration to accommodate the 10× ATAC cell barcode in the i5 piece of the read. Libraries were sequenced to a target of 30,000–35,000 reads/cell as previously recommended.^19^ Raw.bcl files were converted into per-sample.fastq files using Illumina bcl2fastq.

Initial processing of mtscATAC-seq data was performed using the CellRanger-ATAC Pipeline v.2.0.0 by mapping scATAC-seq reads with cellranger-atac count to the GRCh38 reference genome, hardmasked for regions that would otherwise interfere with mapping to the mitochondrial genome (as previously detailed^17^). The outputs included fragments files for downstream epigenomics analyses and.bam files for mtDNA genotyping.

Mitochondrial genotyping was performed on these fragment files with mgatk in tenx mode using the barcodes identified as cells by CellRanger (i.e., cells passing ATAC filter). Only cells with at least 10× coverage of the mitochondrial genome were included in the analysis, achieving a median coverage of 30×-70× across experiments. Across all quality-controlled cells, we observed a mean percell mitochondrial genome coverage of 37.8, with a mean coverage of 39.2 among cells assigned to clones used for downstream clonal analyses.

#### Profiling and validation of mtDNA variants via single-cell TCR

For one ovarian donor (SU-*O*-002), excess CD45^+^ tumor and peripheral blood mononuclear cells were available for additional profiling to corroborate mtDNA-derived clonotypes. To achieve this, single cells were partitioned into GEMs (gel bead-in-emulsions) on a Chromium Controller (10× Genomics; Single Cell 5′ kit, Cat. PN-1000695) following the manufacturer’s protocol with TCR profiling. Reverse transcription was performed in-GEM, followed by emulsion breakage, cDNA cleanup, and amplification. Separate gene-expression and TCR libraries were constructed per 10× recommendations. Library quality and size distributions were verified by TapeStation. Pooled libraries were sequenced on an Illumina Novaseq 6000 instrument for 151 × 151 R1 and R2 read lengths. Raw.fastq files were processed using CellRanger v8 default parameters for both gene expression and VDJ quantification to the hg38 reference.

For downstream analyses, we used mgatk to generate a per-cell, per-mutation heteroplasmy matrix.^19^ Analyzing 5′ scRNA-seq data for mtDNA mutations incurs several limitations as only ∼5% of the mitochondrial genome is transcribed and well-covered by this kit^17^ and the high error rate of mitochondrial transcription leads to many more spurious mutations.^16^ Hence, we restricted our analyses to a subset of high-quality cells with an overall coverage of 20× at well-covered sites detected from the 5′ kit and containing valid TCRs and high-quality gene expression data (i.e., at least 1,000 UMIs and 500 distinct genes detected). For these cells, we considered TCRs with at least 3 cells and performed a supervised Mann-Whitney statistic to determine mutations enriched within a specific TCR, which is the same workflow that we previously used for Smart-seq2 data for T cells.^16^ Figure S1D shows the 10 mutations with the highest statistic where all cells with a mutation detected and a valid TCR were plotted. As many T cells may not be individually barcoded by mtDNA and only ∼5% of the mitochondrial genome can be profiled with the 5′ kit, we specifically do not report overlap statistics but use these data to illustrate the concordance of mtDNA variation with orthogonal clonal markers.

#### scATAC-seq QC, dimensionality reduction and clustering

CellRanger output fragment files were loaded and converted to Arrow files using the createArrowFiles function in ArchR. Quality control metrics were computed for each cell, and only cells with TSS enrichments greater than 4 were kept for all samples. Cells were also filtered based on the number of unique fragments sequenced using a cut-off of 1000. Doublet scores for all cells were computed using the ArchR functions addDoubletScores with k = 10, knnMethod = “LSI”, and LSIMethod = 1.

Sample groupings were defined based on cell types, tissue sites, and disease status (e.g., all cells, lung tumor and NILT samples from patients with NSCLC in Figure 1; myeloid cells, all samples from patients with NSCLC in Figure 3). An ArchR project was then created for each of the sample groupings and doublets were filtered with filterDoublets with a filter ratio of 1. For each ArchR project, dimensionality reduction was performed with addIterativeLSI using default parameters to embed ATAC-data in latent semantic indexing (LSI) space. Next, clustering was performed with addClusters using default parameters.

#### Annotation of mtscATAC-seq dataset

An iterative clustering approach was used to annotate cells, where after each round of clustering, select clusters with relatively high epigenetic similarity (e.g., T and NK cells) are merged and reclustered to achieve desired granularity and higher clustering accuracy. For the NSCLC lung tumor/NILT data sample grouping and ovarian tumor sample grouping, clusters were annotated based on gene score of known marker genes, including *EPCAM*, *KRT18* (epithelial and tumor cells), *VWF*, *PECAM1* (endothelial cells), *COL1A2*, *FBLN1* (fibroblasts), *CD3D*, *CD4*, *FOXP3*, *CD8A*, *KLRD1*, *NCR1* (T and NK cells), *MS4A1*, *PAX5* (B cells), *TNFRSF17*, *VOPP1* (plasma cells), *CD14*, *LYZ* (monocytes), *APOC1*, *CD163* (macrophages), *HLA-DQA1*, *ZBTB46* (DCs), *CLEC4C* (PDCs), *TPSAB1*, *KIT* (mast cells).

9 initial clusters in the lung tumor/NILT data annotated as epithelial or tumor cells were grouped and reclustered, resulting in 22 clusters. 9 clusters were annotated as tumor for satisfying the following criteria 1) highly patient specific and 2) enriched over 4-fold in tumor compared to NILT. The other 13 clusters were annotated as lung epithelial subtypes based on high gene score of following signatures *AGER*, *PDPN*, *CLIC5* (alveolar type 1), *SFTPB*, *SFTPC*, *SFTPD*, *MUC1*, *ETV5* (alveolar type 2), *FOXJ1*, *TUBB1*, *TP73*, *CCDC78* (ciliated), *KRT5*, *KRT17*, *MIR205HG* (basal), *MUC5B*, *SCGB1A1*, *BPIFB1*, *PIGR*, *SCGB3A1* (secretory).

6 initial clusters in the lung tumor/NILT data annotated as T/NK cells were further divided into 19 clusters. 5 initial clusters in the ovarian tumor data annotated as T/NK cells were further divided into 13 clusters. These T/NK clusters were annotated using known marker genes, including *CD3D* (broad T), *CD8A* (CD8^+^ T), *CD4* (CD4^+^ T), *GNLY*, *NCR1* (NK/ILC), *FOXP3* (Treg). To support the marker gene-based annotation, cells were projected to human PBMC reference data using the Azimuth application.^84^ The Azimuth-predicted cell types were dominated by CD4^+^ T, CD8^+^ T, Treg, NK, and ILC, which are all associated with distinct clusters and consistent with the mark-gene based annotations. One cluster in the lung data is marked by the gene score pattern of *CD3D*+, *CD8A*−, *CD4*^−^ , *CD56*^+^ and high gene score of NK signature (*GNLY*, *PRF1*, *GZMB*, *KLRB1*, *CCL3*, *KLRF1*, *NCR1*), which was defined as invariant NKT cells.

For the myeloid reclustering annotation, given that DCs comprised a minor fraction of myeloid cells in peripheral blood relative to lung tumors and NILTs (4.4% vs. 27.4%, respectively), we were unable to fully resolve DC subtypes in blood and thus grouped circulating DCs as a single population.

#### Gene signature scoring

Gene scores for individual genes are computed as implemented in ArchR. When computing the composite gene score for a gene signature, gene scores of individual genes in the signature were *Z* score normalized across all cells, and each cell is then scored by taking the mean z-scaled gene scores in the gene signature.

#### Peak calling and motif analysis

For the NSCLC myeloid epigenetic analysis, peak calling was performed as implemented in ArchR. Both level1 (monocyte, macrophage, DC) and level 2 (e.g., CD14^+^ monocyte, DC3, MoMΦ1, etc) cell type annotation was used as grouping in addReproducible-PeakSet() to identify regulatory elements associated with both broad myeloid cell types as well as subtypes. Differential peak analysis was performed using getMarkerFeatures. TF motifs enriched in peak sets are identified using peakAnnoEnrichment. Per-cell TF motif activities were calculated using chromVAR.^82^

#### Mitochondrial genotyping and clone calling

By default, mgatk calls high-confidence heteroplasmic variants by selecting variants with strand correlation>0.65 and variance-mean ratio>0.01. In this study, most donors have multiple samples from different tissue sites. Given that the default mgatk filters are already conservative, a union approach to selecting high-confidence heteroplasmic variants is employed to increase sensitivity, where if a variant passes the default mgatk filters in any sample, then it is considered a valid, informative variant in all other samples from the same donor (e.g., if a variant is determined to be high quality in the lung tumor sample, it is unlikely to be spurious if it is also observed in the peripheral blood sample from the same donor). We noted one polymorphic and highly homologous region (CCCTCCC in GRCh38 chrM:307–314) that led to spurious connections under the relaxed filtering criteria and explicitly disregarded variants from this region. This mitochondrial variant processing procedure for clone calling outputs a cell by variant heteroplasmy matrix combining all samples for each donor and is implemented in the mitotrek.processing module.

Clones are then called for each donor using mitotrek.core.assign_cell_to_clones. Variants present in >20% cells from a donor (likely technical or homoplasmic) or less than 3 cells are removed. The heteroplasmy matrix is then binarized with a cutoff of 0.07 based on the rationale that exact heteroplasmy levels are not reliable given the stochasticity from mitochondrial genome distribution during cell division and variance in per-cell mitochondrial genome coverage. The heteroplasmy cutoff value is chosen based on benchmarking experiments using published data (Figures 1C and 1D). Although our filtering strategy effectively removes variants that are most likely to represent technical artifacts or broadly distributed homoplasmic mutations, it also reflects an inherent trade-off between accuracy and sensitivity.

To convert mitochondrial variants to clones, an undirected weighted graph is constructed in which vertices are variants and edges are defined by the Pearson correlation coefficient between two variants across cells, computed from the binarized heteroplasmy matrix. After removing edges with weights less than 0.5, each connected component in the graph was treated as a distinct clonotype. Most resulting connected components contained only one variant, whereas highly correlated variants (i.e., frequently co-occurring in cells) were grouped into one connected component. For clones defined by a single variant, any cell positive for the associated variant in the binarized heteroplasmy matrix is assigned to the clone. For clones defined by multiple variants, a cell is required to be positive for all associated variants. Finally, cells assigned to multiple clones are discarded.

#### Benchmarking Mitotrek clone assignment

Full-length scRNA-seq via the Smart-seq2 technology of single-cell derived colonies from two donors published by Ludwig et al.^16^ was downloaded as.fastq data from the Gene Expression Omnibus (accession GSE115214). Mitochondrial genotyping was performed using mgatk and cells with at least 100× mitochondrial genome coverage were retained for downstream analysis. Clones were called using mitotrek.core.assign_cell_to_clones with a binarization cutoff threshold set from 0.01 to 0.15. For each ground truth clone label (established in the experimental protocol by physical separation of the clones), the predicted clone label was determined by taking the mode of called clones among cells in the ground truth clone to compute accuracy, which is implemented in mitotrek.core.clone_calling_accuracy.

#### Clone sharing analysis

We consider two mutually exclusive sets of cells *A* and *B* (e.g., two cell types). There are N clones *c*_1_,*c*_2_, .,*c_N_* containing at least one cell in *A*∪*B*. Note that each clone *c_i_* belongs to a set *P_j_* which contains all clones detected in donor *j*. We summarize the clone counts in the two groups as a *N* × 2 matrix *X* where *X_i_*_,1_ and *X_i_*_,2_ correspond to number of clone *c_i_* cells in *A* and *B*, respectively. The fraction of clones shared between the two groups is computed as.


$$\frac{\sum_{C_{i}}X_{i,1}\times X_{i,2}}{\sum_{P_{j}}D_{j,1}\times D_{j,2}}\text{where}D_{j,1}=\sum_{C_{j}}X_{j,1}\text{and}D_{j,2}=\sum_{C_{j}}X_{j,2}\;\text{for}\;\text{all}\;{\text{C}}_{j}\in P_{j}$$

The numerator computes the number of observed cell pairs between two groups that share a clone. The denominator is a normalization factor that represents all possible cell pairs between two groups, accounting for only cell pairs within the same donor, since cross-donor cell pairs are not valid.

The fraction of shared clonotypes provides a more sensitive and robust measure than correlation coefficients, which can be disproportionately influenced by outliers, capturing relative clone sharing while appropriately accounting for the number of cells detected in each cell type.

#### Comparison of clones using binomial generalized linear model with logit link

To account for differences in clone size when comparing clonal lineage biases, we analyzed clonal proportions using a binomial generalized linear model (GLM) with a logit link. For each clone, the number of cells of a given lineage was modeled as a binomial outcome, with the total number of cells assigned to that clone used as the binomial denominator. This framework weights clones by their size, such that larger clones contribute proportionally more information than smaller clones, while testing for differences in underlying lineage bias between conditions or groups. Unless otherwise noted, all statistical comparisons of clonal proportions reported in the manuscript are based on this binomial GLM–based inference.

#### Corroboration via transcriptomic analyses

Using a subset of 45,524 cells profiled from lung tumors and non-involved tissues from Leader et al.,^30^we defined the major differentiation trajectories for CD14^+^ monocytes to DC3s or monocyte-derived macrophages using Slingshot^83^ (Figures S11M–S11O). From this embedding, we could characterize 1,708 DC3-biased and 1,854 macrophage-biased cells that were annotated as CD14^+^ monocytes from the periphery (defined as a 2-fold biased pseudotime for either lineage; Figure S11P). Differential expression analyses between these putative fate-biased monocytes were performed using the FindMarkers function in Seurat with default parameters. These tran- scriptomic analyses add further support to the clonal relationships that we described using mtscATAC-seq, where monocytes adopt a fate-biased epigenomic program, including in the periphery, and the cell state changes can be verified via transcriptional changes.

### QUANTIFICATION AND STATISTICAL ANALYSIS

Statistical analysis of single-cell sequencing data was performed in python (v3.9.4) and R (v4.3.5). Statistical analysis of flow cytometry data was performed in GraphPad Prism (v9.0).

## Supplementary Material

Supplemental Information

Table S1

Table S2

Table S3

Supplemental information can be found online at https://doi.org/10.1016/j.ccell.2026.05.006.

## Figures and Tables

**Table:** KEY RESOURCES TABLE

REAGENT or RESOURCE	SOURCE	IDENTIFIER
Antibodies		
Anti-human CD45 V500 (clone HI30)	BD Biosciences	Cat# 560779; RRID: AB_1937324; Lot 7172744
Anti-human CD3 FITC (clone OKT3)	Invitrogen	Cat# 11-0037-41; RRID: AB_2533240; Lot 2007722
Biological samples		
Fresh human lung tumor and NILT	Stanford Tissue Procurement Shared Resource	N/A
Fresh human ovarian tumor	Stanford Tissue Procurement Shared Resource	N/A
Human peripheral blood mononuclear cells (PBMC)	This paper	N/A
Critical commercial assays		
Chromium Next GEM Single Cell ATAC Reagent Kits v1.1	10× Genomics	Cat# PN-1000209
Chromium Single Cell 5′ kit	10× Genomics	Cat# PN-1000695
Nuclei Isolation for Single Cell ATAC Sequencing	10× Genomics	CG000169
Deposited data		
Raw and processed mtscATAC-seq and mtDNA mutation calls	This paper	GEO: GSE302113
Software and algorithms		
Mitotrek	This paper	https://github.com/vincent6liu/mitotrek
mgatk	Lareau et al.^17^	https://github.com/caleblareau/mgatk
ArchR	Granja et al.^45^	https://github.com/GreenleafLab/ArchR
CellRanger-ATAC Pipeline v.2.0.0	10× Genomics	https://support.10xgenomics.com
chromVAR	Schep et al.^82^	https://github.com/GreenleafLab/chromVAR
Slingshot	Street et al.^83^	https://github.com/kstreet13/slingshot
Seurat	Hao et al.^84^	https://satijalab.org/seurat/
Azimuth	Hao et al.^84^	https://azimuth.hubmapconsortium.org/

## Data Availability

The ArchR analysis software for epigenetic analysis of scATAC-seq data is available on GitHub (https://github.com/GreenleafLab/ArchR). The mgatk software for processing sequencing data for single-cell mitochondrial variant calling is available on GitHub (https://github.com/caleblareau/mgatk). The Mi- totrek software developed in this work for clone calling using single-cell mitochondrial variant data is available on GitHub (https://github.com/vincent6liu/mitotrek). Raw sequencing and processed chromatin accessibility and mtDNA mutation calls are available at the Gene Expression Omnibus accession **GSE302113**. Any additional custom code used for computational data processing and analysis is available from the authors upon request.

## Highlights

- mtDNA lineage tracing enables clonal analysis of innate immune cells in tumors
- Tumor myeloid cells are clonally linked to circulating monocyte precursors
- DC3s derive from monocytes and retain monocytic epigenetic features
- Monocyte fate bias is epigenetically programmed before tumor infiltration
