# Supplementary material for: Clonal lineage tracing of innate immune cells in human cancer
Source: Cancer Cell. Author manuscript; Available in PMC 2026 Jun 27. (PMC13309241; doi:10.1016/j.ccell.2026.05.006)
Supplement: Supplemental Information [file NIHMS2184724-supplement-Supplemental_Information.pdf]

Cancer Cell, Volume *44*

Supplemental information

Clonal lineage tracing of innate immune cells in human cancer

Vincent Liu, Katalin Sandor, Patrick K. Yan, Zhuang Miao, Yajie Yin, Robert R. Stickels, Andy Y. Chen, Kamir Hiam-Galvez, Jacob Gutierrez, Wenxi Zhang, Sairaj M. Sajjath, Raeline Valbuena, Steven Wang, Bence Daniel, Leif S. Ludwig, Brooke E. Howitt, Caleb A. Lareau, and Ansuman T. Satpathy

Supplemental information for

Clonal lineage tracing of innate immune cells in human cancer

Vincent Liu[1-3], Katalin Sandor[2-3], Patrick K. Yan[2-3], Zhuang Miao[1-3], Yajie Yin[2-3], Robert R. Stickels[2-3], Andy Y. Chen[2-4], Kamir Hiam-Galvez[2-3], Jacob Gutierrez[5], Wenxi Zhang[2-3], Sairaj M. Sajjath[6], Raeline Valbuena[1], Steven Wang[1], Bence Daniel[2-3], Leif S. Ludwig[7,8], Brooke E. Howitt[2], Caleb A. Lareau[5], Ansuman T. Satpathy[2-3,9-10]

[1]Department of Genetics, Stanford University, Stanford, CA 94305, USA
[2]Department of Pathology, Stanford University, Stanford, CA 94305, USA
[3]Center for Immunotherapy Design, Stanford University, Stanford, CA 94305, USA
[4]Department of Bioengineering, Stanford University, Stanford, CA 94305, USA
[5]Computational and Systems Biology Program, Memorial Sloan Kettering Cancer Center, New York, NY 10065, USA
[6]Robin Chemers Neustein Laboratory of Mammalian Cell Biology and Development, Howard Hughes Medical Institute, The Rockefeller University, New York, NY 10065, USA
[7]Berlin Institute of Health at Charité – Universitätsmedizin Berlin, 10178 Berlin, Germany
[8]Max-Delbrück-Center for Molecular Medicine in the Helmholtz Association (MDC) Berlin Institute for Medical Systems Biology (BIMSB), 10115 Berlin, Germany
[9]Parker Institute for Cancer Immunotherapy, San Francisco, CA 94129, USA
[10]Lead Contact

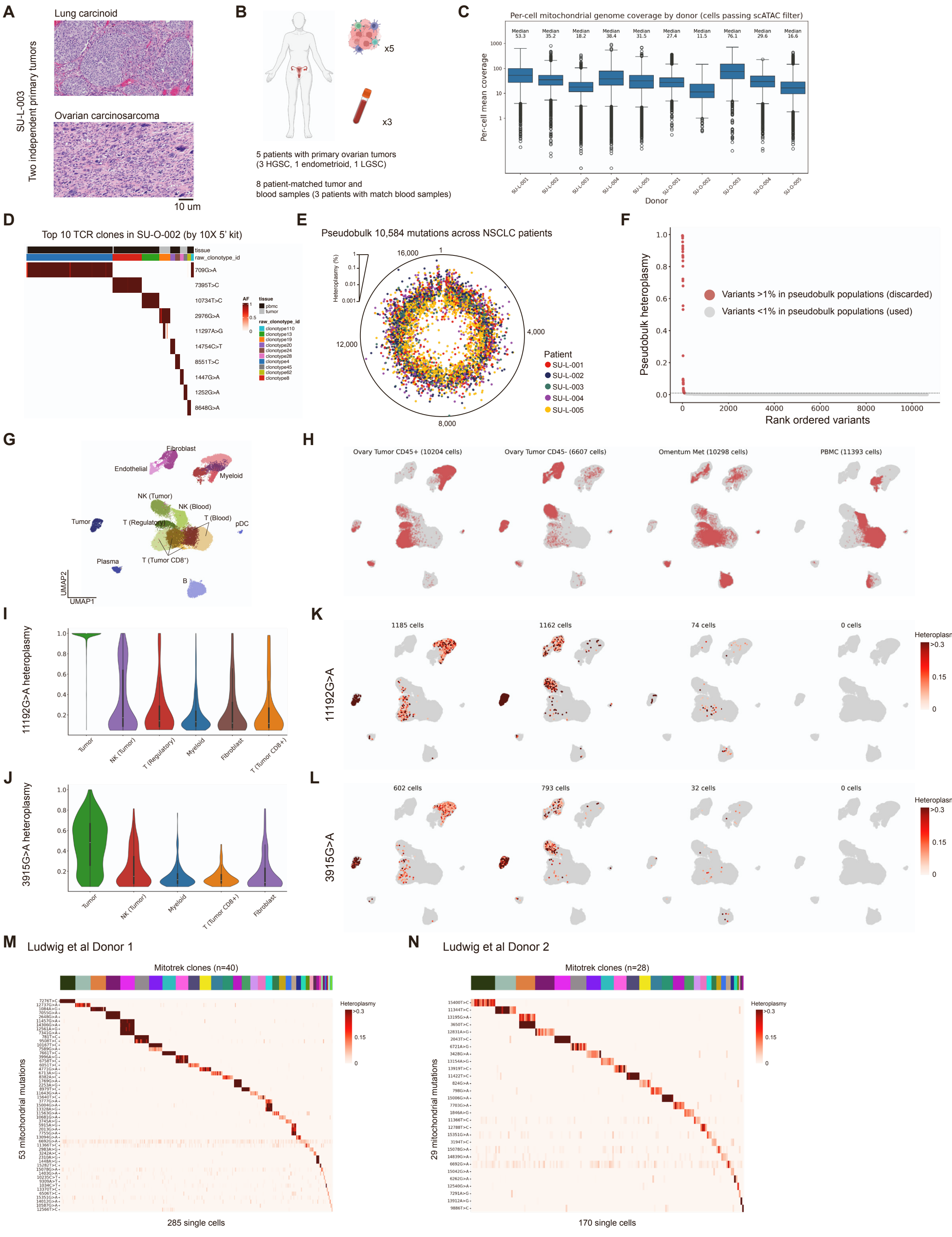

Figure S1: Benchmarking Mitotrek using ground-truth data, Related to Figure 1

(A) Digital pathology scans of patient SU-L-003. Top: scan of low-grade lung carcinoid tumor. Bottom: scan of high-grade ovarian carcinoma.

(B) Schematic summarizing patient and sample information for the ovarian tumor data.

(C) Mitochondrial genome coverage across patients. Boxplots: center line, median; box limits, first and third quartiles; whiskers, 1.5× interquartile range.

(D) Validation of mtDNA clonotypes in T cells by TCR sequencing. Shown in the heatmap are individual cells among the 10 most abundant clonotypes with the heteroplasmy of 10 mutations enriched in a specific TCR (all heteroplasmy is plotted), showing high clonal specificity.

(E) Distribution of mgatk-nominated variants along the mitochondrial genome, averaged across cells and colored by patient.

(F) Pseudobulk heteroplasmy for all variants detected by mgatk across all samples. Variants with >1% pseudobulk heteroplasmy are excluded from downstream analysis. 1% is chosen as the conservative threshold after observing the overall heteroplasmy distribution.

(G) UMAP embeddings of tumor-infiltrating immune cells from matched primary (ovarian) and metastatic (omentum) tumors, and PBMCs from HGSC patient SU-O-005.

(H) Distribution on the UMAP of cells from indicated samples. Cells from the primary ovarian tumor were sorted by CD45 to separate tumor-infiltrating immune cells.

(I) Heteroplasmy levels of 11192G>A, a tumor-specific mitochondrial variants in tumor-infiltrating immune cells processed together with tumor cells.

(J) Same as (I) but for 3915G>A.

(K and L) Mutations projected onto UMAP embeddings across samples. Tumor-specific variants are indiscriminately detected at lower heteroplasmy levels in all cells from the same sample, suggesting mitochondrial transfer and/or technical artifacts (ambient mtDNA).

(M and N) Heatmap showing the heteroplasmy levels of variants (rows) that are identified as clone markers to group cells (columns) in each Mitotrek clone. Position of each variant and the base pair change are shown.

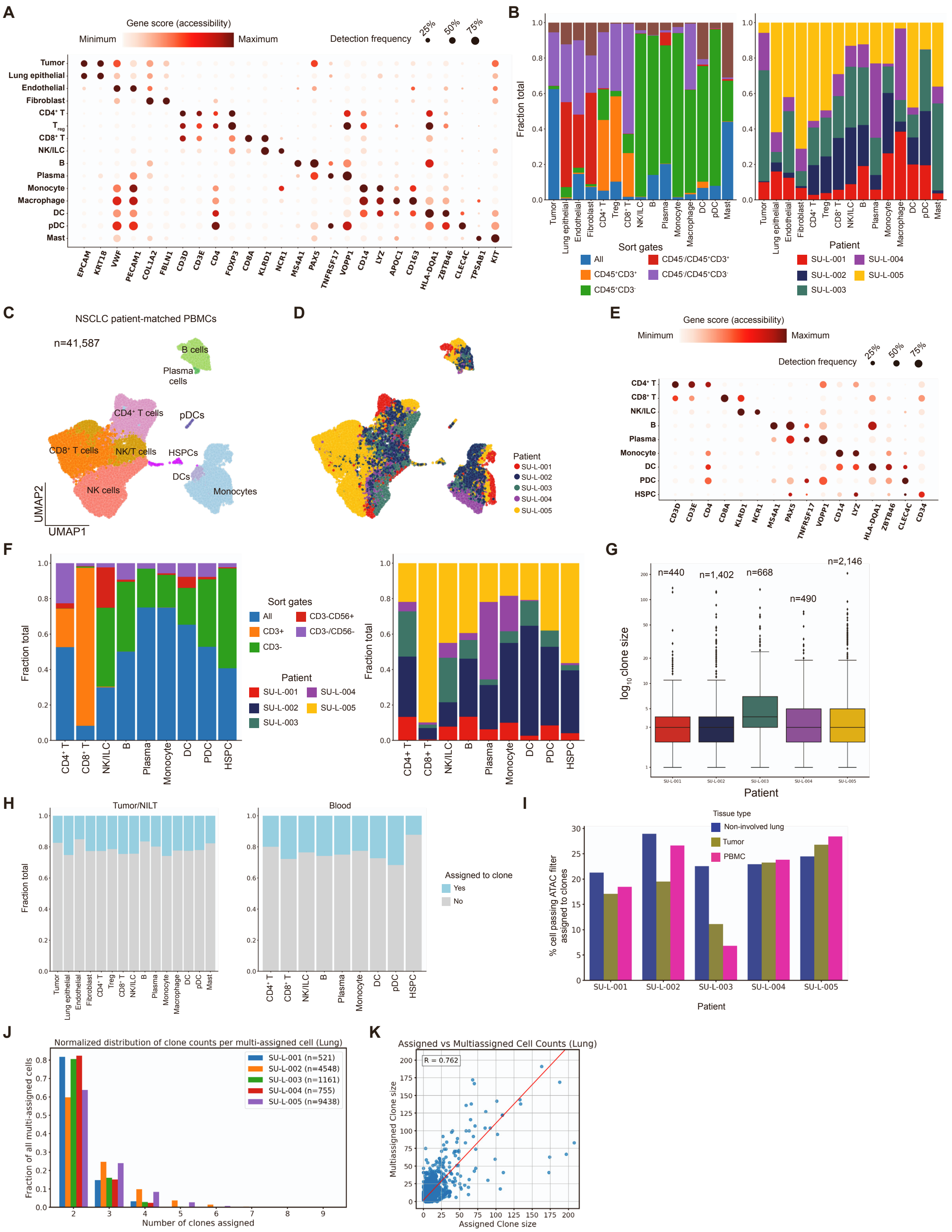

Figure S2: NSCLC data summary and cell type annotation, Related to Figure 1

(A) For cell types annotated in tumor and NILT samples, column-scaled gene accessibility scores and detection frequencies for the indicated genes.

(B) For tumor and NILT samples, bar plots indicating (left) relative proportions of markers used for sorting that were detected in each cell type (certain samples were not sorted, and select sorted samples were merged during single-cell capture, due to sample-specific considerations to optimize single-cell yield) and (right) relative proportions of cells from each patient detected in each cell type.

(C) UMAP of 41,587 PBMCs from patients with lung tumors.

(D) UMAP of cells colored by patient identity.

(E) For cell types annotated in PBMC samples, column-scaled gene accessibility scores and detection frequencies for the indicated genes.

(F) For PBMC samples, bar plots indicating (left) relative proportions of markers used for sorting that were detected in each cell type (certain samples were not sorted, and select samples were additionally sorted using CD56 to enrich innate lymphocytes and myeloid cells) and (right) relative proportions of cells from each patient detected in each cell type.

(G) Clone size distributions for patients with NSCLC. Boxplots: center line, median; box limits, first and third quartiles; whiskers, 1.5× interquartile range.

(H) Bar plots summarizing relative proportions of cells assigned to clones across cell types in tumor/NILT and blood. No significant cell-type bias was observed.

(I) Fraction of cells passing ATAC filters that are successfully assigned to clones.

(J) Characterization of multi-assigned clones in lung tumors. Bar plot showing the distribution of the number of clones assigned for multi-assigned cells.

(K) Clone size comparison between excluded multi-assigned cells and uniquely assigned cells used for downstream analysis. Statistic and P value from Spearman correlation.

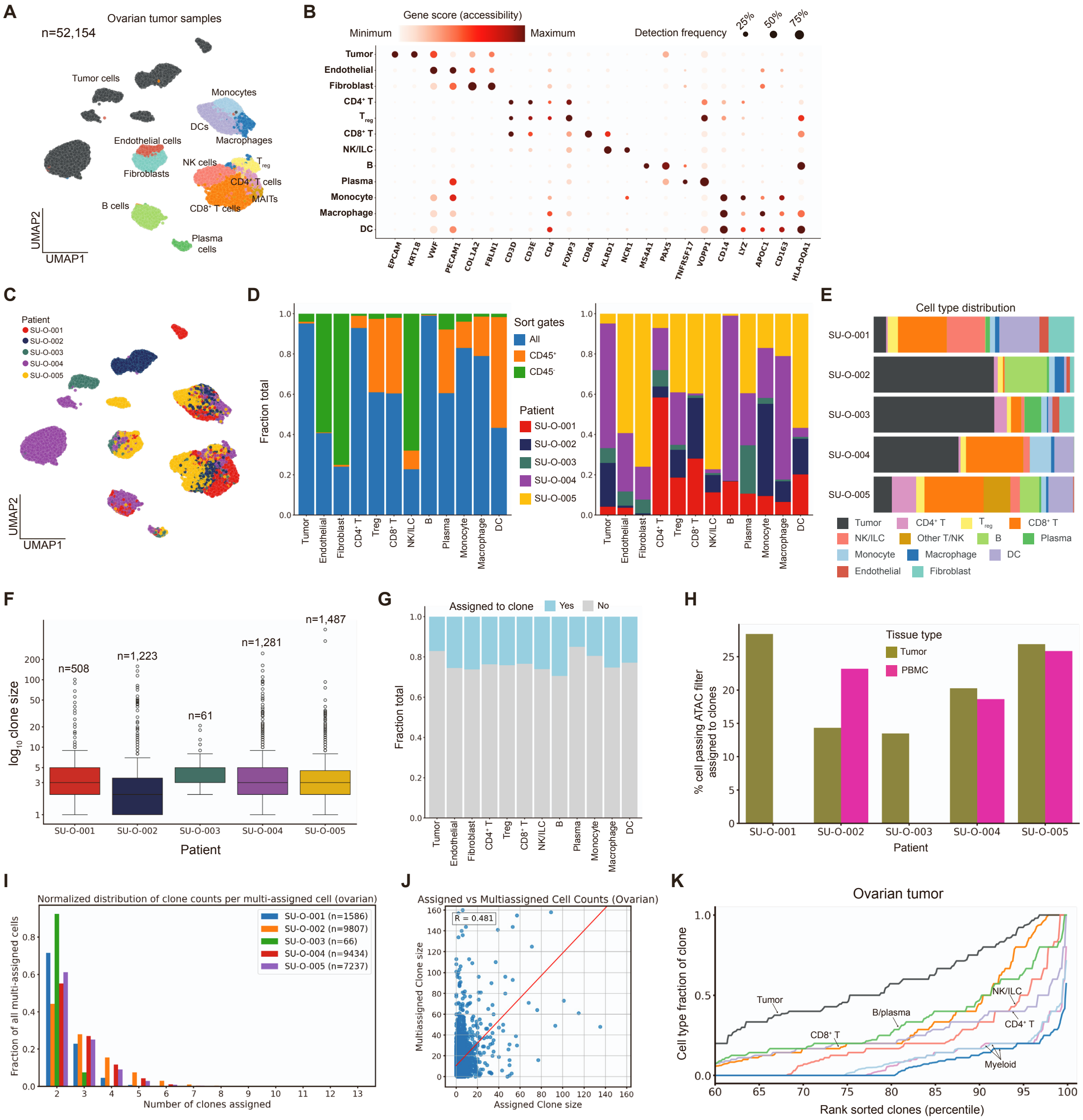

Figure S3: Ovarian cancer data summary and cell type annotation, Related to Figure 2

(A) UMAP of 52,154 cells in ovarian tumors. Cell types denoted by color are inferred after iterative sub-clustering of each of the myeloid, lymphoid, and stromal compartments.

(B) For cell types annotated in ovarian tumor samples, column-scaled gene accessibility scores and detection frequencies for the indicated genes.

(C) UMAP of cells colored by patient identity.

(D) For ovarian tumor samples, bar plots indicating (left) relative proportions of markers used for sorting that were detected in each cell type (certain samples were not sorted, and select sorted samples were merged during single-cell capture, due to sample-specific considerations to optimize single-cell yield) and (right) relative proportions of cells from each patient detected in each cell type.

(E) Normalized bar plot showing cell type composition for each patient.

(F) Clone size distributions for patients with ovarian cancer. Boxplots: center line, median; box limits, first and third quartiles; whiskers, 1.5× interquartile range.

(G) Bar plots summarizing relative proportions of cells assigned to clones across cell types in ovarian and blood. No significant cell-type bias was observed.

(H) Fraction of cells passing ATAC filters that are successfully assigned to clones. Peripheral blood samples were obtained from three patients.

(I) Characterization of multi-assigned clones in ovarian tumors. Bar plot showing the distribution of the number of clones assigned for multi-assigned cells.

(J) Clone size comparison between excluded multi-assigned cells and uniquely assigned cells used for downstream analysis. Correlation test: Spearman.

(K) Cumulative fractions of clones stratified by cell type for cells from ovarian tumor samples. Clones with ≥5 cells are considered for this analysis.

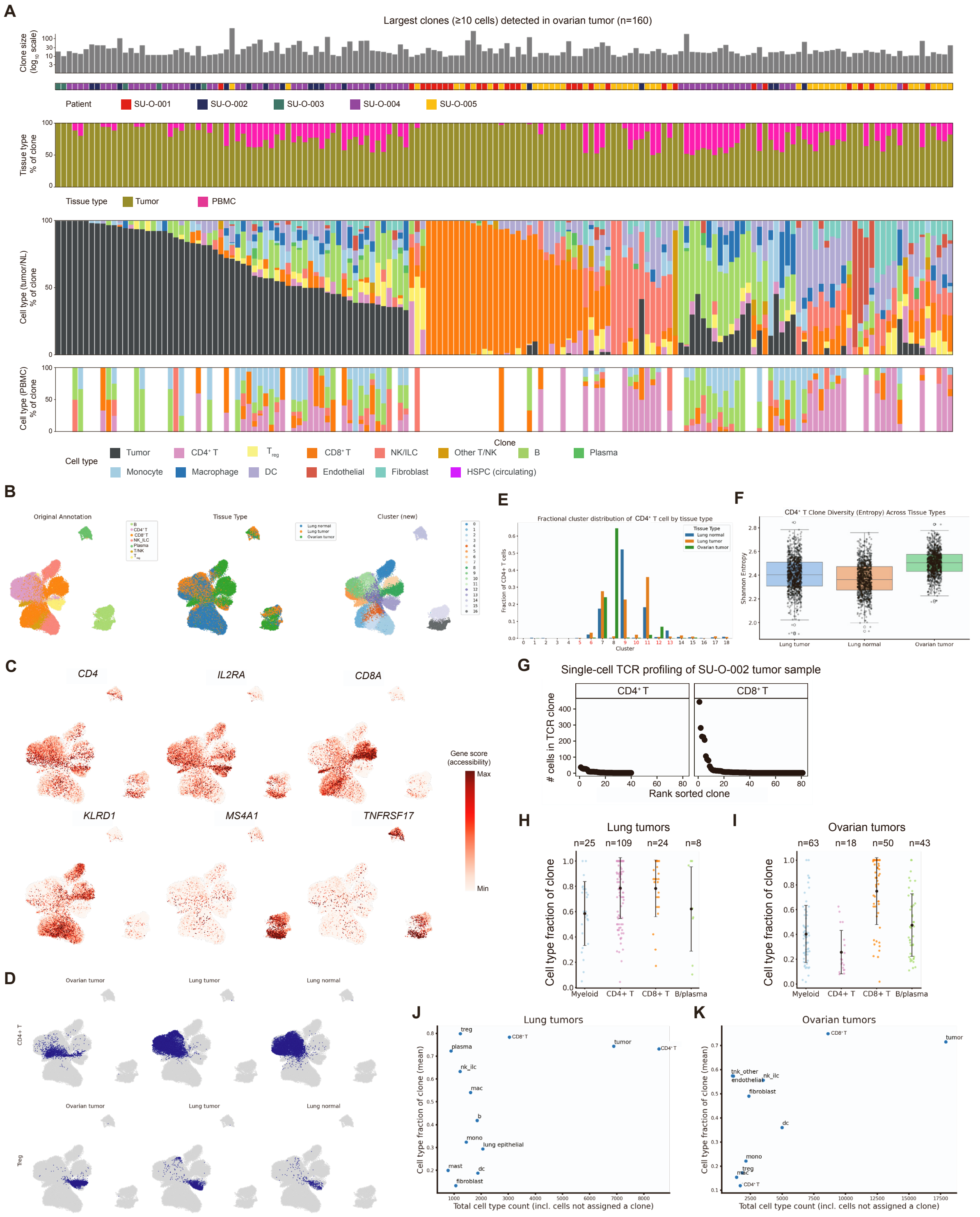

Figure S4: Additional clonal landscape analyses, Related to Figure 2

(A) All clones with at least 10 cells detected in ovarian tumor samples. Each column represents a unique clone.

(B) UMAP of lymphocytes in all solid tissue samples, including both lung and ovarian tumors.

(C) Accessibility of lymphocyte markers distinguish them on UMAP.

(D) Visualization of $CD4^+$ T and Treg stratified by tissue type on UMAP, showing overlap by tissue type and supporting the validity of the original cell type annotation.

(E) Cluster distribution of originally labeled $CD4^+$ T cells.

(F) $CD4^+$ T-cell clonal diversity across tissue types. Shannon entropy was computed from clone frequencies after restricting the analysis to the seven $CD4^+$ T clusters. For each tissue type, 500 cells (including both assigned and unassigned clones) were repeatedly sampled with replacement (1,000 iterations) to normalize for cell number and quantify clonal diversity. Boxplots: center line, median; box limits, first and third quartiles; whiskers, 1.5× interquartile range. No statistical test is performed.

(G) Distribution of scTCR-seq derived clone sizes for $CD4^+$ and $CD8^+$ T cells from SU-O-002. Clones were defined using CellRanger vdj and plotted for clonotypes with at least 2 cells observed.

(H) Annotation of lung tumor clones. Clones with ≥5 cells of the indicated cell type are considered, and the distribution of the indicated cell type's clone fraction is plotted. For myeloid cells, monocytes, macrophages, and DCs are grouped. For $CD4^+$ T cells, T_{reg}s and other $CD4^+$ T cells are grouped. n represents the number of clones considered for each indicated cell type.

(I) Same as (H) but for ovarian clones.

(J) Relationship between cell type abundance and mean clone fraction in lung tumors. Mean clone fraction and total number of cells are plotted for each cell type, showing no correlation.

(K) Same as (J) but for ovarian tumors.

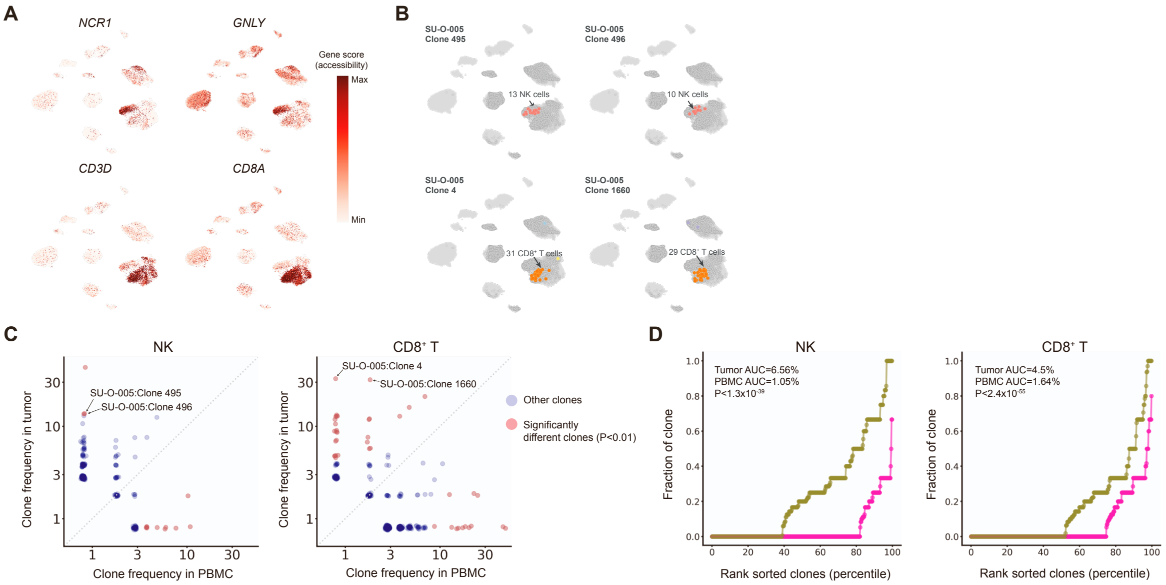

Figure S5: A case report of tumor-specific clonal expansion, Related to Figure 2

(A) Accessibility of NK and CD8$^+$ T markers distinguish them on UMAP.

(B) Representative clones capturing clonal expansion events of NK cells (top row) and CD8$^+$ T cells (bottom row) in SU-O-005. For each clone, cells from the clone's donor are highlighted with shaded circles, and cells assigned to that clone are colored by their cell type.

(C) Scatterplots comparing clone frequencies of circulating cells with those infiltrating the tumor. The largest clones are locally expanded and minimally detected in the periphery. Significance is determined by Benjamini-Hochberg adjusted Fisher's exact test.

(D) Cumulative fraction of clone sizes for the indicated lymphoid cell types, split by tissue site. AUC corresponds to the overall clone size for the indicated cell type and tissue site. P values are reported from a Wald statistic from binomial GLM.

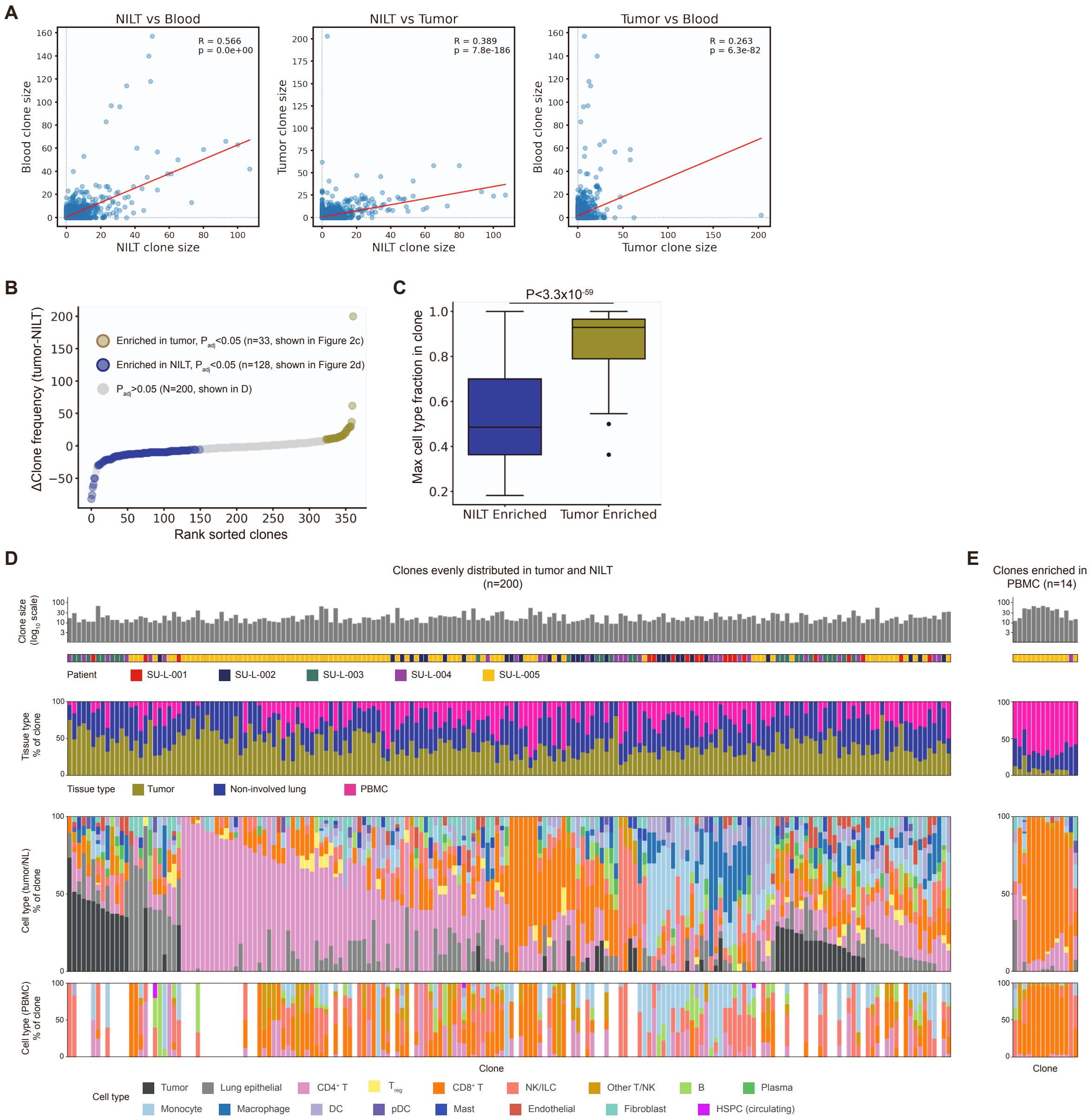

Figure S6: Comparative clonal analysis across tissue sites in NSCLC, Related to Figure 2

(A) Summary of clone Spearman correlations between anatomical sites. Clone sizes are significantly correlated between tissue sites.

(B) Enrichment of clones in NILT or tumor. Significance is determined by Benjamini-Hochberg adjusted Fisher's exact test against overall tissue site distribution for clones with at least 10 cells in tumor and NILT.

(C) Comparison of dominant cell type fraction distribution between clones enriched in NILT and in tumor. Boxplots: center line, median; box limits, first and third quartiles; whiskers, 1.5× interquartile range. P value from binomial GLM.

(D) Clones enriched in neither tumor nor NILT, as determined by p value > 0.05 from Benjamini-Hochberg adjusted Fisher's exact test against overall tissue site distribution for clones with at least 10 cells in tumor and NILT. Each column represents a unique clone.

(E) Clones enriched in PBMC from the same analysis shown in (D).

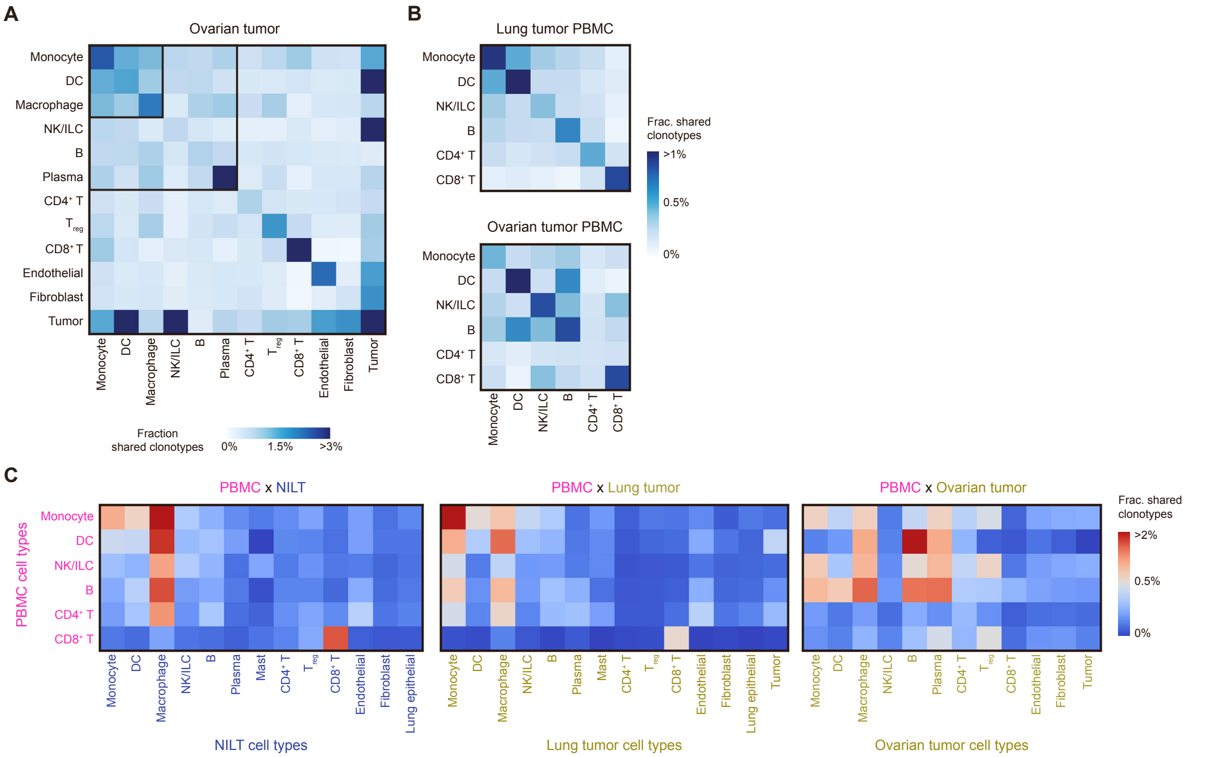

Figure S7: Additional cross-tissue clonal analysis, Related to Figure 2

(A) Heatmaps showing the fraction of all cell pairs belonging to the same clone and consisting of two cell types within ovarian tumor. Pairs were restricted to cells from the same donor.

(B) Same as in (A) but for PBMCs.

(C) Heatmap showing the fraction of all cell pairs belonging to the same clone and consisting of a PBMC cell type and a solid tissue cell type.

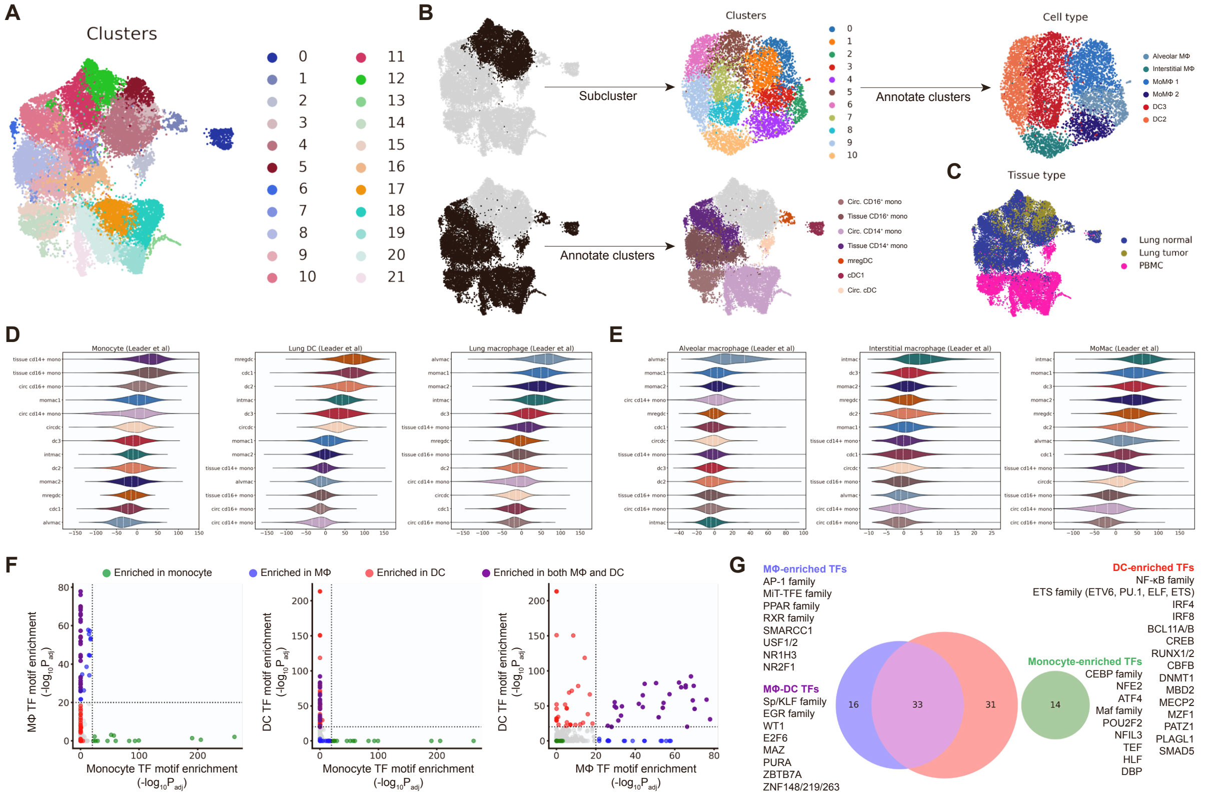

Figure S8: NSCLC myeloid annotation and epigenetic analysis, Related to Figure 3

(A) UMAP of myeloid cells from PBMC, lung tumor, and NILT samples of patients with NSCLC, colored by their original cluster assignments.

(B) Myeloid annotation scheme. Monocyte clusters separated clearly into CD14$^+$ and CD16$^+$ subsets, which were annotated without further subclustering. Macrophages and DCs were subclustered to achieve higher granularity and annotated based on subclustering results.

(C) UMAP of cells colored by tissue sites.

(D) Gene scores of published signatures derived from single-cell RNA-seq data.

(E) Same as (D) but for proteomic data.

(F) TF motifs enriched in the marker peaks of three major MNP cell types. P-values are calculated from the Benjamini-Hochberg adjusted Wilcoxon signed-rank test.

(G) Summary of TFs whose motifs are enriched in broad MNP cell types. A $-\log_{10}P_{adj}>20$ cutoff was used.

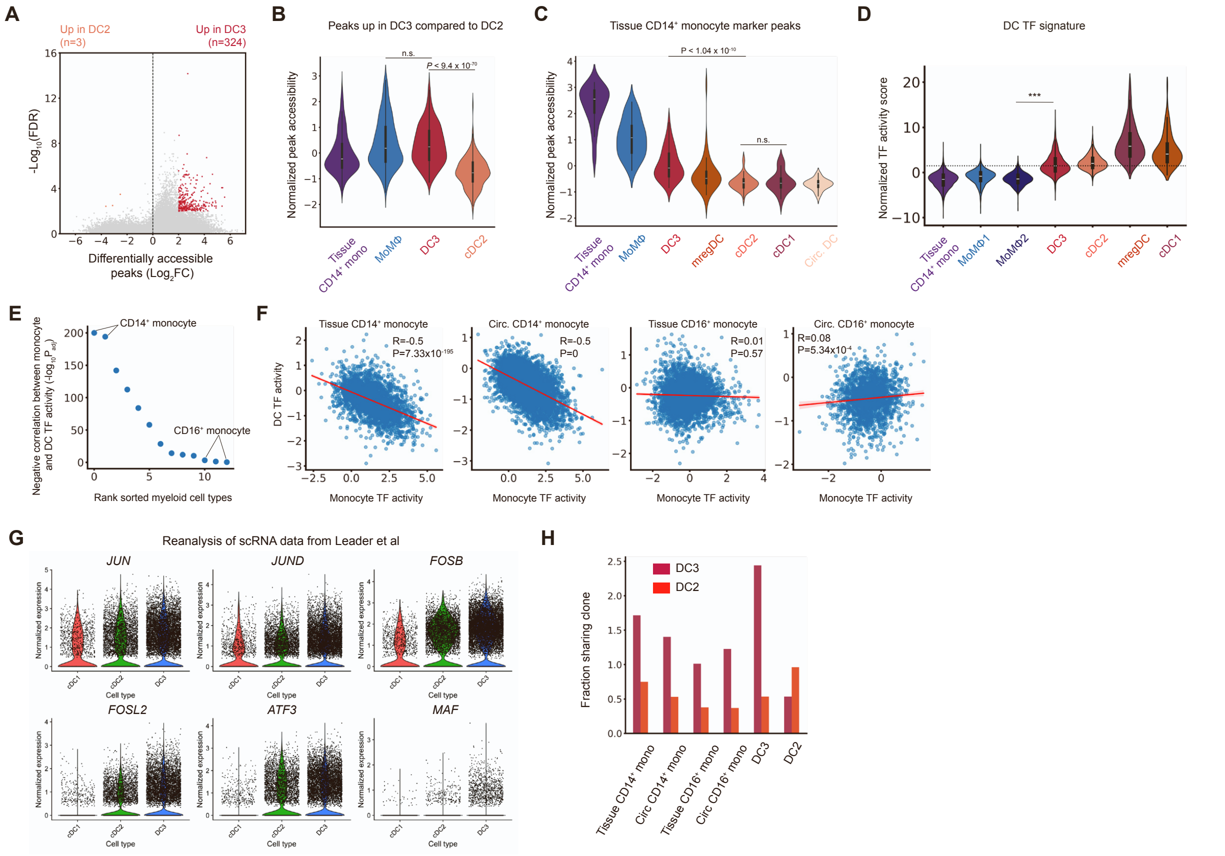

Figure S9: DC3 epigenetic and clonal analysis, Related to Figure 3

(A) Differentially accessible genomic regions in DC3 vs DC2.

(B) Normalized sum accessibility of genomic regions significantly more accessible in DC3 compared to DC2 in indicated cell types. P-values are from a Mann-Whitney U test.

(C) Normalized sum accessibility of genomic regions significantly more accessible in CD14+ monocytes compared to other myeloid subtypes. P-values are from a Mann-Whitney U test.

(D) Average chromVAR motif deviation scores for DC TFs highlighted in **Figure 3C**. Kruskal-Wallis test.

(E) Statistical significance of monocyte-DC TF motif accessibility correlation in all myeloid subtypes. $CD14^+$ monocytes in tissue and circulation display the most significant negative correlation between monocyte and DC TF motif accessibilities.

(F) Monocyte and DC TF activities are negatively correlated only in $CD14^+$ monocytes but not in $CD16^+$ monocytes. Correlation test: Spearman.

(G) RNA expression of monocyte-associated TFs in DC subtypes. scRNA-seq data and annotation are procured from a prior report.

(H) Monocytes share more clonotypes with DC3 than with DC2.

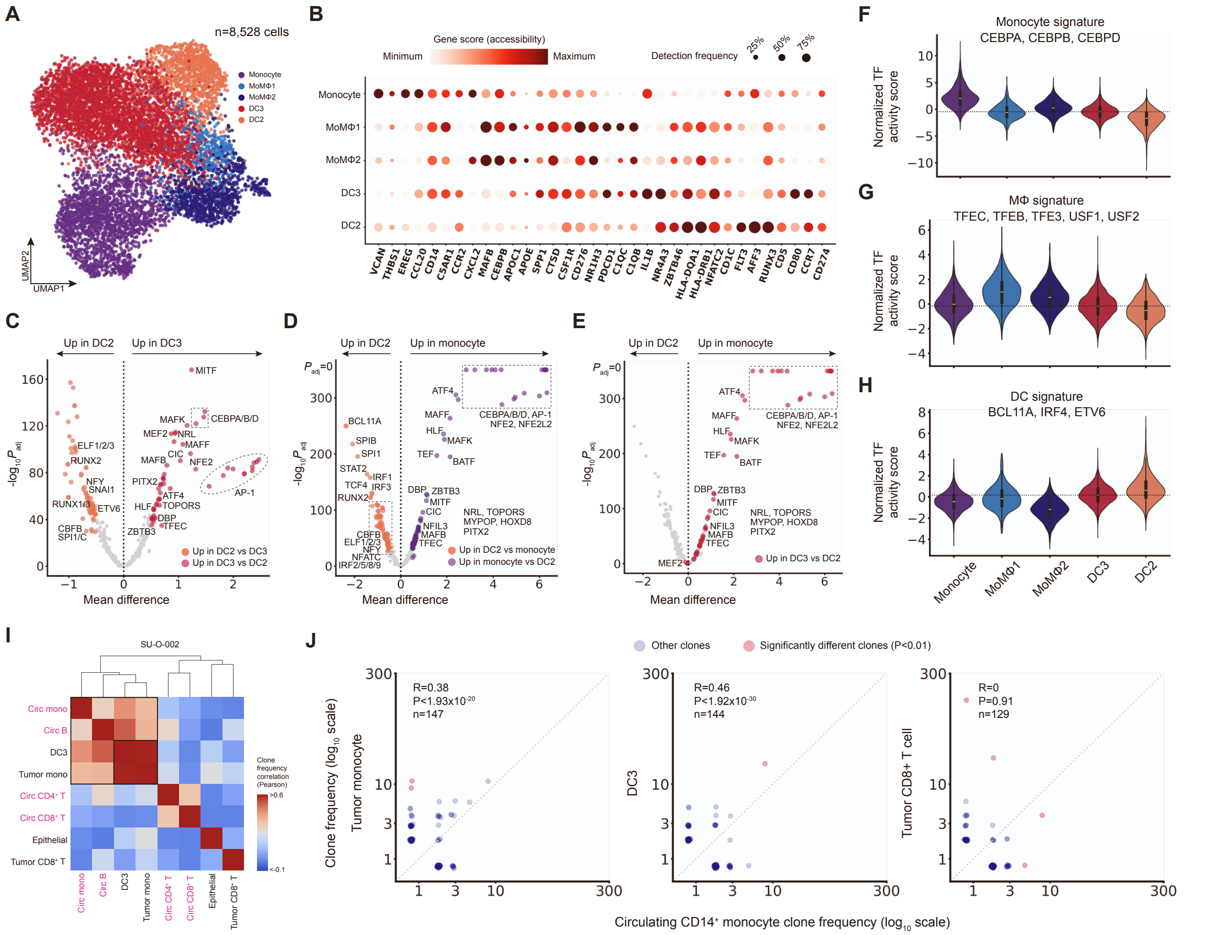

Figure S10: Ovarian myeloid epigenetic analysis, Related to Figure 3

(A) UMAP of myeloid cells from ovarian tumor samples.

(B) Column-scaled gene accessibility scores and detection frequencies for the indicated genes.

(C) Differentially active TF motifs between DC2 and DC3. P-values are calculated using the Benjamini-Hochberg adjusted Kruskal-Wallis test.

(D) Same as (C) but for DC2 and $CD14^{+}$ monocytes

(E) Same as (D) but colored by TFs up in DC3 vs DC2 shown in (C).

(F) Average chromVAR motif deviation scores for the indicated monocyte-associated TFs.

(G) Same as (F) but for macrophage-associated TFs.

(H) Same as (F) but for DC-associated TFs.

(I) Cell type-cell type clone frequency correlation across clones (≥5 cells across all samples). Color denotes correlation value, computed using Pearson's ρ. Text labels of circulating PBMC cell types are colored pink.

(J) Scatterplots comparing clone frequencies of circulating $CD14^+$ monocyte and tissue $CD14^+$ monocyte (left), DC3 (middle), and tumor $CD8^+$ T cell (right). Significantly different clones with $P<0.05$ adjusted Fisher's exact test are highlighted red. Correlation test: Spearman.

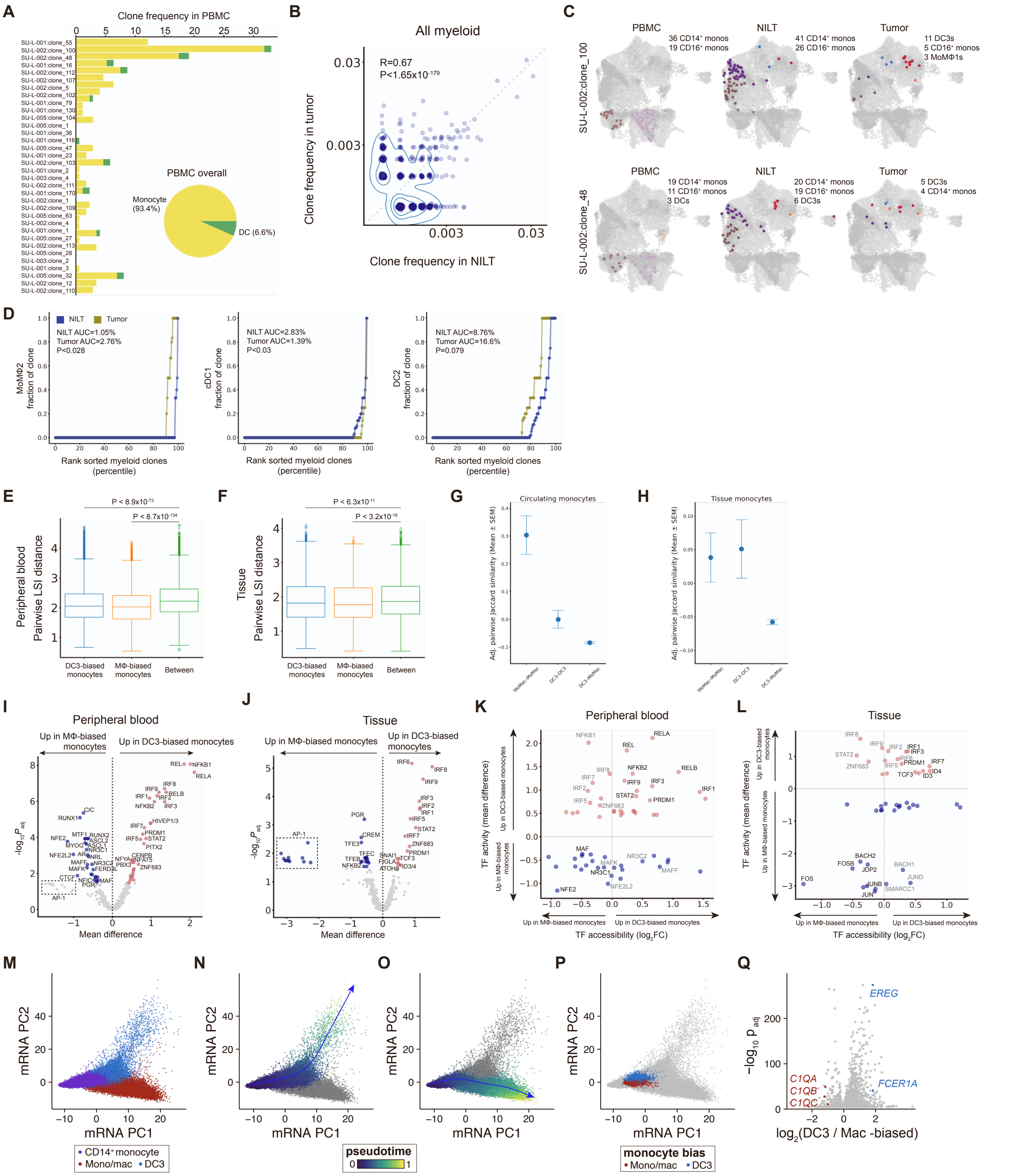

Figure S11: Epigenetic comparison of DC- and macrophage-biased myeloid clones, Related to Figure 4

(A) Monocyte and DC proportions of largest myeloid clones (≥10 cells) in PBMC samples of patients with NSCLC.

(B) Scatterplots comparing clone frequencies of myeloid cells in tumors with those in NILT. Contours visualize density. Correlation Test: Spearman.

(C) Representative clones capturing myeloid cell type distribution across tissue sites. For each clone, cells from the clone's donor are highlighted with shaded circles, and cells assigned to that clone are colored by their cell type.

(D) Cumulative fraction of clone sizes for the indicated myeloid cell types, split by tissue site. AUC corresponds to the overall clone size for the indicated cell type and tissue site. P-value from binomial GLM.

(E) Epigenetic similarity as measured by distance in the LSI space within and between DC3-biased and macrophage-biased monocytes in circulation. Statistic Kruskal-Wallis test.

(F) Same as (E) but for tissue-resident cells.

(G) Normalized genome-wide similarity among monocyte fate-biased subpopulations of monocytes in circulation. Genome-wide chromatin accessibility similarity was computed using the Jaccard index after binarizing 500-bp cell-by-bin matrices and normalizing raw values to correct for sequencing-depth–dependent inflation, following Fang et al.. Shown are mean ± SEM adjusted pairwise similarities for macrophage-biased, DC-biased, and cross-group monocyte pairs in circulation.

(H) Same as (G) but for tissue-derived monocytes.

(I) Differentially active TF motifs between DC3-biased and macrophage-biased monocytes in peripheral blood. P-values are calculated using the Benjamini-Hochberg adjusted Kruskal-Wallis test.

(J) Same as (I) but for tissue-resident monocytes.

(K) Joint comparison of TF gene body accessibility and inferred genome-wide TF activity to nominate TFs driving differential fate outcomes of monocytes in circulation

(L) Same as (K) but for tissue-resident monocytes.

(M) Reduced dimensionality of 45,524 cells from prior lung profiles colored by cell cluster.

(N) Pseudotime trajectory of DC3 differentiation from scRNA-seq profiles.

(O) Same as (N) but for monocyte-derived macrophages.

(P) Fate-biased monocytes used for differential expression.

(Q) Differential expression analyses of monocytes biased toward macrophages or DC3s. Selected genes supporting cell state are highlighted.
